# Replication fork integrity and intra-S phase checkpoint suppress gene amplification

**DOI:** 10.1093/nar/gkv084

**Published:** 2015-02-11

**Authors:** Anna Kondratova, Takaaki Watanabe, Michael Marotta, Matthew Cannon, Anca M. Segall, David Serre, Hisashi Tanaka

**Affiliations:** 1 Department of Molecular Genetics, Cleveland Clinic Lerner Research Institute, Cleveland, OH, USA; 2 Department of Surgery, Cedars-Sinai Medical Center, West Hollywood, CA, USA; 3 Genomic Medicine Institute, Cleveland Clinic Lerner Research Institute, Cleveland, OH, USA; 4 Department of Biology, San Diego State University, San Diego, CA 92182, USA

## Abstract

Gene amplification is a phenotype-causing form of chromosome instability and is initiated by DNA double-strand breaks (DSBs). Cells with mutant *p53* lose G1/S checkpoint and are permissive to gene amplification. In this study we show that mammalian cells become proficient for spontaneous gene amplification when the function of the DSB repair protein complex MRN (Mre11/Rad50/Nbs1) is impaired. Cells with impaired MRN complex experienced severe replication stress and gained substrates for gene amplification during replication, as evidenced by the increase of replication-associated single-stranded breaks that were converted to DSBs most likely through replication fork reversal. Impaired MRN complex directly compromised ATM/ATR-mediated checkpoints and allowed cells to progress through cell cycle in the presence of DSBs. Such compromised intra-S phase checkpoints promoted gene amplification independently from mutant *p53*. Finally, cells adapted to endogenous replication stress by globally suppressing genes for DNA replication and cell cycle progression. Our results indicate that the MRN complex suppresses gene amplification by stabilizing replication forks and by securing DNA damage response to replication-associated DSBs.

## INTRODUCTION

Increase in the copy number of genomic segments harboring oncogenes and therapy target genes (gene amplification) causes aggressive tumor phenotypes by promoting cancer progression and acquired therapy resistance (1–4). Defining cellular processes and factors regulating gene amplification would help us to identify molecular targets for controlling aggressive tumors. Experimentally, gene amplification has been shown to be facilitated either by agents that damage DNA or by endonucleases (i.e. I-SceI) that induce DNA double-strand breaks (DSBs) at specific sites (5–9). In tumors, gene amplification is a spontaneous event and is initiated by endogenous DNA breaks. Endogenous breaks in cancer cells can arise from physiological stresses including replication stress, which directly involves DNA synthesis at a very vulnerable time (10,11). Replication stress, the slowing or stalling of replication forks and/or DNA synthesis, can be caused by activated oncogenes and/or insufficiency of DNA precursors (11–13). Stalled replication forks, if not protected, can collapse and be resolved into chromatids with broken ends. Thus, cellular processes/factors that protect stalled forks can also be important regulators of gene amplification.

To prevent DSBs during replication, stalled forks need to restart before collapsing. In simple organisms, homology-dependent recombination (recombination restart) can escort stalled forks to restart before collapsing (14,15). Although the mechanism of the restart in metazoans is less clear, previous studies showed that the DNA repair protein Mre11 could play a role; Mre11 is at replication foci in nuclei (16,17) and mediates the restart of replication inhibitor-induced stalled forks (18). Thus, Mre11/Rad50/Nbs1 (MRN) deficiency could result in collapsed forks and DSBs. The MRN complex is highly conserved, with multiple roles in DSB repair (19–21). The MRN complex is recruited very early to DSBs where it facilitates DNA damage response by checkpoint activation through ATM (22,23) and possibly through ATR (24). The MRN complex, together with CtIP, possesses 3′-to-5′ exonuclease and endonuclease activities that initiate the resection of DSB ends (25,26). The resulting single-stranded DNA (ssDNA) tails search for homology and invade into the sister chromatid for legitimate, homology-directed (HD) repair. Structurally, the MRN complex consists of globular domains (Mre11 and Nbs1) and coiled-coil and hook domains (Rad50) (27,28). The hook domain mediates the homodimerization of the complex and creates a rigid parallel structure with two arms of coiled-coil domain spanning 1000 Å. With the DNA binding activity of the Mre11 globular domain, the dimer could bridge two sister chromatids and assist HD repair of DSBs. These structural properties could also be utilized to hold the sister chromatids together at the stalled forks and facilitate fork restart. Such an important function at stalled forks explains why the MRN complex is essential in cell proliferation (29–31), and could explain the accumulation of DSBs during *in vitro* replication in *Xenopus laevis* egg extracts after Mre11 depletion (32,33).

DSBs formed by replication stress are incompatible with cell proliferation. For example, in precancerous tissues, oncogene-induced replication stress activates p53 that blocks G1/S transition and cell proliferation by inducing apoptosis and senescence (11). Abrogating this barrier by p53 mutations allows cells to proliferate and progress into cancerous states. This is also important for controlling gene amplification, considering that association of loss of p53 function with gene amplification is a well-established fact (34,35). However, p53 loss is necessary but not sufficient for gene amplification; thus, other safeguard mechanisms against gene amplification at different cell cycle stages should exist. In yeast, stalled forks invoke intra-S phase checkpoint through activation of Rad53 kinase (a yeast homologue of Chk2 and functional orthologue of Chk1) (36,37). Rad53, activated by Mec1 (a yeast homologue of ATR), protects forks from collapsing and arrests the cell cycle. In higher eukaryotes, intra-S phase checkpoint also prevents replication fork from collapsing (38,39). ATM senses DSBs, while ATR is activated by ssDNA accumulating at stalled forks (40,41). These kinases phosphorylate the downstream effector kinases Chk1 (mainly ATR) and Chk2 (mainly ATM). The effector kinases, in particular Chk1, maintain replication fork integrity by slowing down DNA synthesis and by inhibiting additional origin firing (42,43). Thus, ATM/ATR-mediated intra-S phase checkpoint could function as an additional safeguard mechanism against gene amplification. Alternatively, ATM/ATR is epistatic to p53 in suppressing gene amplification, as ATM phosphorylates and activates p53 (44).

To study processes and factors that regulate gene amplification, we knocked down Mre11 in a p53-mutant Chinese hamster ovary (CHO) cell line system (Mre11-KD cells). We found that frequency of gene amplification increased 10-folds in Mre11-KD cells. Massive fork collapse during replication and impaired ATM-dependent checkpoint promoted gene amplification proficiency. Importantly, ATR/Chk1-dependent checkpoint was functional in Mre11-KD cells, indicating that Mre11 *per se* is required for preventing fork collapse. Finally, Mre11-KD cells exhibited global transcriptional changes that resulted in the suppression of genes for DNA metabolism including replication initiation. These results demonstrate the important role of Mre11 in maintaining replication fork integrity, failure of which can lead to deleterious phenotypes such as gene amplification proficiency.

## MATERIALS AND METHODS

### Cloning and cell culture

To knockdown *Mre11* in our CHO-dhfr-derivative cell lines, we constructed a vector expressing CHO Mre11 shRNA. shRNA oligos were synthesized and cloned into lentiviral vector pLSLP (45) (pLSLP-CHOshMre11–562). DNA sequences for oligos used in this study are available upon request. pLSLP-CHOshMre11–562 was transfected into 293T cells with two other vectors (pVSV-G and pCMV-delta-8.2) to produce lentiviral particles that were infected into D229IRlox2–35-noIR-2 (D229IRlox2–35-Mre11KD) (9). Infected cells were selected with puromycin to establish a pool of cells with Mre11 shRNA.

A plasmid encoding human MRE11 cDNA (pTP17) (46) was a gift from Dr Tanya Paull (The University of Texas at Austin). Human MRE11 cDNA (hMre11) was cloned into lentiviral expression vector pLV-CMV-neo (pLVneo-TP17). Viral particles were infected into the Mre11KD cells. G418 selection established a pool of cells expressing human MRE11.

### *DHFR* amplification assay

Cells were exposed to methotrexate (MTX) for 12 days and resistant colonies were counted; (i) 10^4^ cells were selected with MTX at the concentration of 0.4 μM and (ii) 10^5^ cells were selected with MTX at the concentration of 0.8 μM. Cell culture media with MTX were changed every 4 days. Colonies were fixed with 1% glutaraldehyde solution (1% glutaraldehyde, 1 mM MgCl_2_, 100 mM NaPO_4_, pH7) and stained with 0.1% crystal violet.

Fluctuation analysis was done from single cell-derived cell populations. Cell populations were first plated onto 96-well plates at very low cell density and clones were isolated from wells that had only one colony. Clones were expanded up to 10^6^ cells and 10^5^ cells were plated onto a 10 cm^2^ plate for MTX selection (0.8 μM).

### FACS

For mitotic shake-off, cells were grown on 225 cm^2^ flasks to semi-confluent density. Flasks were gently tapped for mitotic cells (being at both pre- and post-cytokinesis mitotic stages) to detach from the bottom. Mitotic cells were then plated and analyzed at indicated time points.

For DNA content assessment, cells were trypsinized, centrifuged at 2000 rpm for 5 min, resuspended in 200 ul of medium, fixed with 70% ethanol and kept at −20°C for overnight. Fixed cells were stained with propidium iodide using Propidium Iodide Flow Cytometry Kit (Abcam). FACS was performed by FACSCalibur flow cytometer. Obtained data were analyzed with FlowJo software.

Click-iT® EdU Alexa Fluor® 488 Flow Cytometry Assay Kit (Life Technologies) was used for analyzing DNA replication after treatment with DNA damaging agents. Cells were incubated with several different concentrations of drugs either for 22 h (etoposide) or 4 h (camptothecin, CPT). The last 2 h of incubations were done with EdU.

### Western blotting

Cells were lysed in RIPA Lysis buffer (Millipore) supplemented with 0.1% SDS, protease inhibitor cocktail (Sigma) and phosphatase inhibitor cocktails (Sigma). Samples were prepared using NuPage LDS Sample buffer (Invitrogen) with 100 mM DTT, and proteins were resolved by sodium dodecyl sulphate-polyacrylamide gel electrophoresis using the NuPage MOPS system (Invitrogen). Antibodies used were as follows: anti-Mre11 (Novus Biological, NB 100–142, 1:2000), anti-NBS1 (p95/NBS1, Cell Signaling #3002, 1:1000), anti-Rad50 (Novus Biological, NB 100–154, 1:500), anti-pATM S1981(Cell Signaling #4526, 1:500), anti-pATR S1989 (Kerafast, 1:500), anti-Chk1 S317 (Cell Signaling, #2344, 1:400), anti-Chk1 S345 (Cell Signaling, #2348, 1:400).

### *In situ* immunocytochemistry

Asynchronous (for *γ-*H2Ax and 53BP1 co-staining) or cells synchronized by mitotic shake-off (for *γ-*H2Ax foci counting at different cell cycle stages) were plated, collected at the indicated time points and fixed and stained on 12 mm circular coverslips immersed into wells of 12-well plates. *γ-*H2AX staining was performed according to the manufacturer's protocol (Phospho-Histone H2A.X- Ser139 Antibody #2577, 1:1000, Cell Signaling). For co-staining with 53BP1, cells were incubated with rabbit anti-53BP1 antibody conjugated with Alexa488 (Novus Biological, NB100–904G, 1:200). Foci were counted in three independent series of images, each of which contained 50 cells. Experiments were repeated at least three times.

### COMET assay

Alkaline and neutral COMET assays were performed and analyzed according to the manufacturer's protocol (CometAssay kit, Trevigen). Cells were grown to 30–50% confluence and aphidicolin (2 mM) was added for 20 h. DNA was stained with SYBRGold reagent. DNA intensity for each comet was measured using ImageJ software (supplemented with CometAssay add-in) and Extent Tail Moment (ETM) was calculated. Comets were assigned to different damage groups based on the range of ETM and morphology (Supplementary Figure S4). Neutral conditions include no damage (EXT<50), medium damage (50<EXT<150) and severe damage (EXT>150). Alkaline conditions include no or minor damage (EXT<50, with big round head and virtually no tail), medium damage (50<EXT<250, with round dense head and rounded tail) and severe damage (EXT>250, with big drop-like or small head merged with dense tail), experiments were performed at least twice for each condition and at least 200 cells were analyzed for each condition.

### Transcriptome analysis

Total RNA was isolated from Mre11-KD and control cells (infected with LV-GFP) using RNeasy plus mini kit (QIAGEN). Two microgram of total RNA was processed to construct sequencing libraries using TrueSeq RNA Sample Preparation Kit (Illumina) at the Genomics Core at Cleveland Clinic Lerner Research Institute. Thirty-five base pair, single-end sequencing was done using Genome Analyzer IIx at the Nucleic Acid Shared Resource of Ohio State University. Library construction and sequencing were performed in duplicate for Mre11-KD cells and control cells. The numbers of reads obtained were: 24 504 094 (control-1), 26 689 524 (control-2), 27 404 397 (Mre11KD-1) and 26 288 201 (Mre11KD-2). GEO accession number for the sequences is GSE59487.

Fastq files containing RNAseq data were aligned to the Chinese hamster reference genome (July 2013 (C_griseus_v1.0/criGri1) assembly downloaded from the UCSC database) using Tophat. Gene scaffold data (ref_CriGri_1.0_scaffolds.gff3) was included in the alignment. After alignment, the total number of reads mapping to each exon was counted using custom perl scripts. The edgeR package within R was used to quantify gene expression differences between Mre11-KO and control cells (47). To calculate false discovery rates, we used the q-value R package, which estimates the proportion of false positives based on the overall distribution of *P*-values.

## RESULTS

### Mammalian cells with reduced expression of the MRN complex

We have previously established a clone derived from *DHFR*-deficient CHO cells (D229IRlox2–35-noIR-2) in which a single-copy mouse *DHFR* expression cassette was stably integrated into a chromosome (9). Treatment of these cells with MTX results in selection of resistant clones with amplified *DHFR*; thus, we can measure the frequency of gene amplification by counting resistant colonies. To determine the role of the MRN complex in suppressing gene amplification, we knocked down *Mre11* using a lentiviral vector expressing shRNA against Chinese hamster *Mre11*.

**Figure 1.**
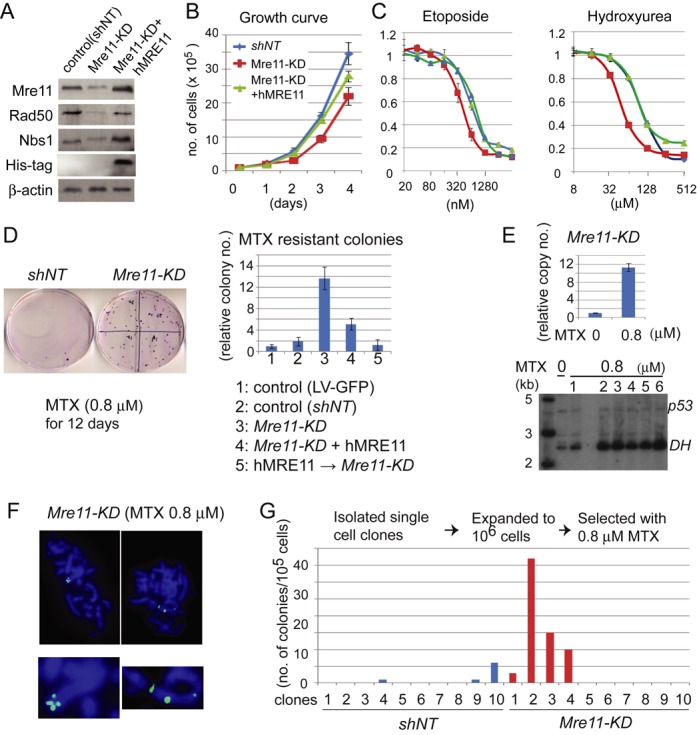
(**A**) Reduced expression of the MRN complex in Mre11-KD cells. β-actin was used as a loading control. Human MRE11 has a HIS-tag on the C-terminus. (**B**) Reduced proliferation of Mre11-KD cells. Growth curves are shown for control cells (shNT, blue), Mre11-KD cells (red) and Mre11-KD cells complemented with human MRE11 (green). (**C**) Mre11-KD cells are hypersensitive to DNA damaging agents. Genotypes are color-coded as in B. Cell survival was measured after four days of treatment. (**D**) MTX-resistant colonies arise more frequently in Mre11-KD cells. *Left*: 0.1% crystal violet-stained plates after 12 days of MTX selection. 10^5^ cells were plated and treated with 0.8 μM MTX for 12 days before fixation and staining with 0.1% crystal violet. *Right*, histograms showing the relative increase in the number of colonies in each genotype (normalized to the number of LV-GFP colonies). (**E**) *DHFR* amplification causes MTX resistance. *Top*: relative increase of copy numbers in the 0.8 μM MTX-resistant Mre11-KD cells, measured by real-time PCR. *Bottom*: Southern blot analysis of *DHFR* amplification in 0.8 μM MTX-resistant Mre11-KD clones. Untreated cells (0 μM MTX) and resistant clones (0.8 μM MTX, 1–6) were co-hybridized with p53 probe and *DHFR* probe. (**F**) Intra-chromosomal *DHFR* amplification. Metaphase spreads were obtained from 0.8 μM MTX-resistant cell populations and probed with fluorophore-labeled *DHFR* cDNA. Whole metaphase nuclei are shown on the top and chromosomes with signals are magnified on the bottom. (**G**) The number of colonies fluctuates among single cell-derived clones. For each genotype, 10 single cell-derived clones were isolated and subjected to MTX selection. Histogram shows the number of MTX-resistant colonies for each clone per 10^5^ cells.

Complete loss of *Mre11* expression leads to severe proliferation defect in vertebrate cells (29–31). Fourteen days after lentiviral infection, we recovered a population of cells with significantly reduced expression of Mre11 protein (Mre11-KD cells), relatively to the control cells expressing either non-targeting shRNA or green fluorescent protein (GFP) (Figure 1A). Other components of the complex (Rad50 and Nbs1) were also reduced, likely due to the fact that the reduction of Mre11 destabilizes the entire complex and leads to proteolytic degradation of other components (48). Expressing human MRE11 (hMre11) in Mre11-KD cells resulted in the increase of both Rad50 and Nbs1 proteins, indicating that human MRE11 can form a stable complex with Chinese hamster Rad50 and Nbs1.

Consistent with the essential role of *Mre11* in cell proliferation, Mre11-KD cells exhibited an impaired growth phenotype. After 4 days of culture, the number of Mre11-KD cells was 60% of that of control cells (Figure 1B). The number was improved to 80% of that of the control cells for Mre11-KD cells expressing human MRE11. The reduced cell number was not due to the increase in cell death, because we did not detect an increase in the sub-G1 cell population by FACS analysis (Supplementary Figure S1A). Mre11-KD cells had increased sensitivity to DNA damaging agents (Figure 1C). Increase of sensitivity was noted for both hydroxyurea (a ribonucleotide reductase inhibitor) and etoposide (a topoisomerase II inhibitor). In both cases, expressing human MRE11 rescued the hypersensitivity.

### Frequent spontaneous, intra-chromosomal gene amplification in Mre11-KD cells

Given the well-established role of DNA damage in the initiation of gene amplification (5–9), we expected that gene amplification could occur more frequently in Mre11-KD cells than in control cells. To determine the frequency of gene amplification, we plated 10^5^ cells and cultured them for 12 days with either 0.8 μM MTX before resistant colonies were counted (Figure 1D, Supplementary Table S1). Indeed, colonies arose at much higher frequency in Mre11-KD cells; on average, the number of colonies was increased 7.2-fold in Mre11-KD cells relative to in cells with non-target shRNA (13.6-fold relative to in cells with lentivirus expressing GFP). This fold increase could still be an underestimate, as Mre11-KD cells grew slower and tended to form smaller colonies than control cells. Ectopic expression of human MRE11 in Mre11-KD cells significantly suppressed the emergence of MTX-resistant colonies. Finally, cells introduced with human MRE11 before knocking down hamster Mre11 completely suppressed gene amplification proficiency.

We confirmed *DHFR* amplification as the cause of MTX resistance (Figure 1E). *DHFR* copy number was increased 11-fold in MTX-resistant cell pools. For individual resistant clones, Southern blotting showed that *DHFR* gene was highly amplified relative to a single-copy control (*p53*). Fluorescence *in situ* hybridization demonstrated that amplified *DHFR* genes were located within a single chromosome in all 64 metaphase spreads examined (Figure 1F, Supplementary Figure S2).

There are two possible scenarios for the observed gene amplification proficiency in Mre11-KD cells. First, *DHFR* amplification can be an MTX-induced event. Inhibition of DHFR by MTX decreases intracellular nucleotide pools, blocks replicative DNA synthesis and results in DNA breaks. Such breaks could initiate *DHFR* amplification as MTX-induced events. Alternatively, Mre11-KD cells can generate spontaneous variants with *DHFR* amplification more often than control cells during normal cell proliferation (without MTX), and these spontaneous mutants are selected by MTX treatment. The fluctuation test (49) can determine whether mutants arise as induced events or spontaneously (Figure 1G and Supplementary Table S2). Ten single cell-derived clones were isolated, expanded and were subject to MTX (0.8 μM) selection. If *DHFR* amplification is an MTX-induced event, roughly the same number of colonies is expected from each clone. If *DHFR* amplification is a spontaneous event, the number of colonies would highly fluctuate; the number should be dependent on when *DHFR* amplification occurred during the expansion of clones prior to MTX selection. Among 10 clones established from Mre11-KD cells, three clones produced a number of resistant colonies (37, 15 and 10 per 10^5^ cells), whereas none of the colonies were seen in six clones. These results indicate a spontaneous nature of *DHFR* amplification during normal cell growth.

### Chromosome instability in Mre11-KD cells

To identify abnormal characteristics during normal cell proliferation that could underlie gene amplification proficiency, we investigated nuclear morphology and DNA content. Three observations were notable. First, large nuclei with increased DNA content (>4 N) were observed more frequently in Mre11-KD cells (4%) compared to control cells (1.8%) (Supplementary Figure S1A). Second, chromatin bridges were more common (3-fold, 2.9% in Mre11-KD cells versus 0.8% in control cells) (Supplementary Figure S1B). Chromatin bridges form when either a chromosome with two centromeres or a chromosome with incompletely replicated DNA is segregating (50). Finally, micronuclei, small chromatin masses comprised of either lagging or broken chromosomes, were also more frequent in Mre11-KD cells (Supplementary Figure S1C) (10% in Mre11-KD cells versus 2.8% in control cells). These observations suggest chromosomal instability of Mre11-KD cells. To assess the dynamic nature of this instability, we examined 25 single-cell-derived colonies after 7–9 cell divisions and evaluated cell number, nuclear size and the number of cells with micronuclei (Supplementary Figure S1D). Nuclei were on average larger and more heterogeneous in both the size and number within each colony of Mre11-KD cells. Furthermore, several colonies carried a large number of micronuclei; nine clones carried more than six cells with micronuclei per colony.

Increased DNA content (i.e. tetraploidization) could facilitate gene amplification, because all the genes become redundant and thus the genome can be modified without the risk of loss of essential genes (51). If tetraploidization precedes *DHFR* amplification, we should observe increased chromosome numbers in MTX-resistant cells. However, the number of chromosomes was similar between untreated and resistant cells (34–38 chromosomes) (Supplementary Figure S3). Some of the metaphases form MTX-resistant cells had only 24–28 chromosomes. Thus, cells with *DHFR* amplification most likely arose directly from cells with the same DNA content as parental cells.

### Increase in both ssDNA and dsDNA breaks during replication in Mre11-KD cells

**Figure 2.**
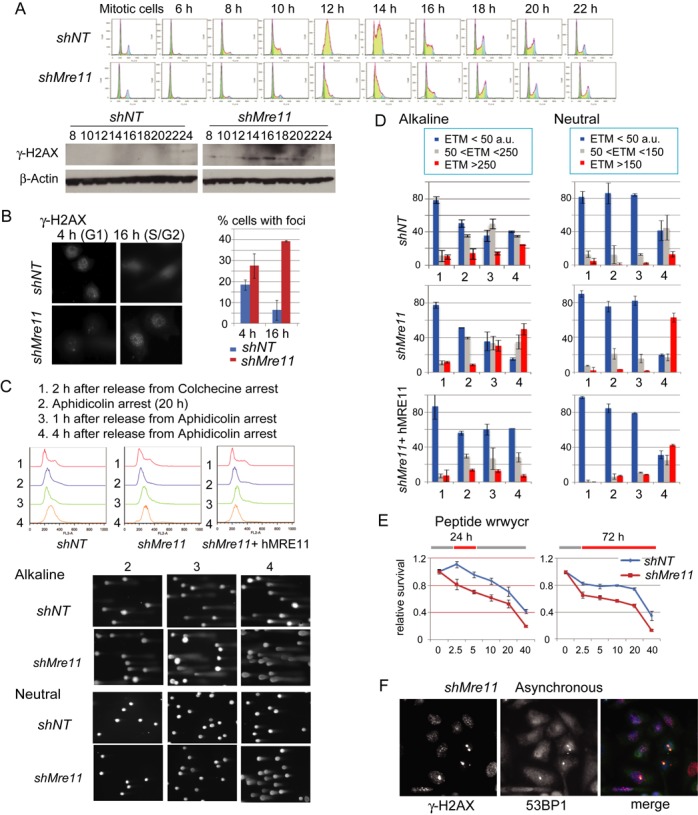
(**A**) Increased γ-H2AX in S-phase in Mre11-KD cells. *Top*: cells were collected by mitotic shake-off, and cell cycle progression was monitored at indicated time points by FACS. *Bottom*: western blotting analyses for γ-H2AX of the same samples. (**B**) Cells with multiple γ-H2AX foci are increased during S-phase in Mre11-KD cells. γ-H2AX immunostaining (*left*) and the percentage of cells with γ-H2AX foci at 4 h (G1) and 16 h (G2) after release (*right*) are shown. (**C**) Single cell electrophoresis (Comet) analysis. Experimental conditions (*top*), FACS profiles for each condition and genotype (*middle*) and representative gel images from control and Mre11-KD cells (*bottom*) are shown. Note that, in alkaline conditions, tail moments increased at 2 h after release from aphidicolin arrest (condition 3), whereas in neutral conditions tail moments increased at 4 h after release (condition 4). (**D**) DNA breaks during replication in Mre11-KD cells. The degree of DNA damage is evaluated as a tail moment. For each genotype, results in alkali (*left*) and neutral conditions (*right*) are shown. The experimental conditions (1, 2, 3 and 4 in X-axis) are indicated in C. Y-axis represents the fractions of cells with the indicated tail moments. (**E**) Mre11-KD cells are hypersensitive to peptide WRWYCR that binds to the four-way DNA junctions. WRWYCR was added to the media 24 h after plating the cells. *Left*: relative cell survival after the 24-h treatment followed by the 48 h of recovery without WRWYCR; *right*: relative cell survival after 72 h treatment with WRWYCR. (**F**) Co-localization of large γ-H2AX foci and 53BP1 nuclear bodies in post-mitotic cells. Nuclei from asynchronous Mre11-KD cells were stained with anti- γ-H2AX and anti-53BP1 antibodies. Post-mitotic (G1 phase cells) can be distinguished by their small size and paired localization.

Abnormal nuclear morphology can also reflect defects in DNA replication (50). We synchronized cells by mitotic shake-off (mechanical detachment by agitation of loosely attached cells undergoing mitosis from the adherent cell culture) and identified time points when the majority of cells were either in G1 (4–8 h) or in S (10–16 h) phase (Figure 2A). We first examined the phosphorylated histone H2AX (γ-H2AX), a marker for DSBs and stalled replication forks, by western blotting (Figure 2A, bottom). In contrast to the control cells, γ-H2AX gradually increased in S-phase from 12 h to 16 h in Mre11-KD cells. This increase in protein was correlated with the increase of cells with multiple γ-H2AX foci in S-phase: at 16 h 38% of cells exhibited multiple, small foci, compared with only 6% in control cells (Figure 2B). We also noticed that in G1 (at 4 h), Mre11-KD cells (and to a lesser extent control cells) contained cells with a few, large γ-H2AX foci that were different from the small foci in S-phase (see below and Figure 2F).

We directly determined DNA lesions causing the S-phase specific increase of γ-H2AX. We examined both single-strand breaks (SSBs) and DSBs using the single cell gel electrophoresis assay (Comet assay). In Comet assay, broken, smaller DNA migrates away as the tails of the comet from undamaged, highly organized DNA in the heads of the comet by electrophoresis (Figure 2C, Supplementary Figure S4). Electrophoresis in alkaline (denaturing) gels can detect both SSBs and DSBs, whereas in neutral gels only DSBs can be detected. We used the ETM (defined as tail length x%DNA in the tail) as a measure of broken DNA (52) (Figure 2D). In G1 cells at 2 h after release from a colchicine block (referred to as cell population 1), roughly 80% of nuclei had very small ETMs (ETM<50, in blue) in both control and Mre11-KD cells. In cells arrested at early S-phase by the DNA polymerase inhibitor aphidicolin (cell population 2), nuclei with medium damages (in gray, 50<ETM<250 a.u. for an alkaline condition and 50<ETM<150 a.u. for a neutral condition) were increased in both control and Mre11-KD cells in alkaline condition, indicating that inhibiting DNA polymerase resulted in stalled forks, ssDNA gaps and SSBs. Resuming replication by releasing cells from aphidicolin block generated more SSBs and DSBs in Mre11-KD cells than control cells, indicating more stalled forks in Mre11-KD cells. At 1 h after release (cell population 3), Mre11-KD cells with severe damages (in red, ETM>250 a.u. for an alkaline condition and ETM>150 a.u. for a neutral condition) increased only in an alkaline condition, whereas at 4 h after release (cell population 4), such cells became abundant in both conditions. Therefore, SSBs preceded DSBs in Mre11-KD cells, strongly suggesting that stalled replication forks collapsed into broken forks. In contrast, <15% of nuclei had severe damages in control cells (in neutral conditions). Ectopic expression of human MRE11 (hMre11) in Mre11-KD cells partially rescued cells from severe damages; <40% of total nuclei showed severe damages at 4 h after release.

### Stalled and collapsed (broken) forks in Mre11-KD cells

Two experiments support the notion that stalled replication forks collapsed into broken forks after the release from aphidicolin block in Mre11-KD cells. First, checkpoint abrogation increased DSBs. In both yeast and human cells, an intra-S phase checkpoint stabilizes stalled forks and thus prevents fork collapse and breaks (36–39). We cultured cells with the ATM/ATR inhibitor caffeine, isolated DNA in agarose plugs and separated broken DNA from chromosomal DNA by pulse field gel electrophoresis (Supplementary Figure S5). Caffeine treatment increased smaller species of DNA (broken DNA) in Mre11-KD cells, but not in control cells. Second, we evaluated stalled/broken fork intermediate, Holliday junction-like four-way junctions (reversed forks) (53,54). Reversed forks could be more abundant in Mre11-KD cells than in control cells. To test this possibility, we measured the sensitivity to Holliday junction-binding peptide WRWYCR (55–57) (Figure 2E). WRWYCR binds to the center of four-way junctions *in vitro*, traps junctions inside living cells and causes cell death. We examined the sensitivity either after 24 or 72 h treatment. In both experiments, Mre11-KD cells were significantly more sensitive to WRWYCR than control cells.

Having abundant stalled and broken forks within a cell, Mre11-KD cells may have difficulty in completing replication before entering mitosis. We noticed a few, large γ-H2AX foci during G1 phase (Figure 2F): such foci could represent regions of under-replicated DNA. This can be addressed by immunostaining with 53BP1. 53BP1 forms large foci during M and G1 phase (53BP1 nuclear bodies) that co-localize with γ-H2AX at under-replicating regions (58,59). We found that 53BP1 nuclear bodies in G1 nuclei of Mre11-KD cells co-localized frequently with γ-H2AX foci and were often symmetrical between two daughter nuclei. These results indicate problems in completing replication in Mre11-KD cells.

### Mre11-KD cells activate both ATR and ATM in response to replication inhibitors, but not to DSBs

**Figure 3.**
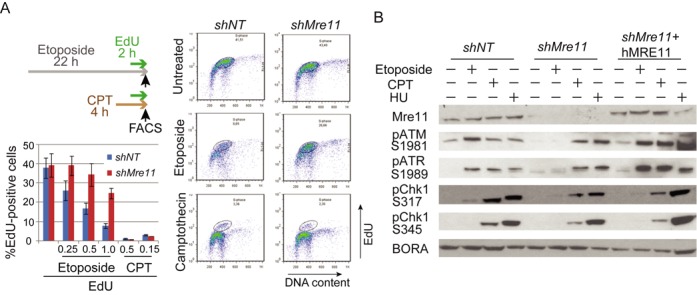
(**A**) Activation of intra-S and G2/M checkpoint in response to DNA damaging agents. Checkpoint activation was compromised in response to etoposide, but not to the replication inhibitor CPT in Mre11-KD cells. Experimental designs (*top left*): cells were treated either etoposide (22 h) or CPT (4 h). EdU was added for 2 h before FACS analyses. *Right*: profiles of cells with EdU incorporation for untreated cells (*top*), cells treated with etoposide (*middle*) and cells treated with CPT (*bottom*). Circles indicate cells with replicating DNA (labeled with EdU). *Left bottom*: histograms of EdU-positive cells. Several concentrations of agents were used. Note that EdU-positive cells are more abundant in Mre11-KD cells than in control cells when treated with etoposide, but not with CPT. (**B**) Activation of intra-S and G2/M checkpoint kinases in response to DNA damaging agents. Mre11-KD cells lack activation of ATM, ATR and Chk1 checkpoint kinases in response to etoposide, but not to replication inhibitors. Cells were treated as indicated, and activating phosphorylation of kinases was monitored by western blotting. Staining for Bora was used as a loading control.

The abundant stalled and broken forks in S-phase can activate intra-S and G2/M cell cycle checkpoints. However, although at a decreased rate (Figure 1B), Mre11-KD cells proliferated well, suggesting that checkpoints were to some extent impaired. To test this possibility, we treated cells with etoposide for 24 h, then labeled with 5′-ethynyl-2′-deoxyuridine (EdU) and analyzed the EdU incorporation by FACS (Figure 3B). The fraction of EdU-positive cells represent cells failed to arrest upon etoposide treatment and continued DNA replication. Without etoposide, similar fractions (45–50%) of cells were EdU-positive in control and Mre11-KD cells. However, with increasing concentrations of etoposide, Mre11-KD cells escaped from arrest more often than control cells. For example, at the maximal dose of 1 mM etoposide, 25% of Mre11-KD cells were EdU-positive whereas only 7% of control cells were EdU-positive. In contrast, Mre-11 KD cells arrested as efficiently as control cells in response to CPT (Figure 3A). EdU-positive cells were equally absent after CPT treatment in both Mre11-KD cells and control cells, indicating that cell cycle progression was efficiently blocked by replication inhibitors.

To investigate the molecular basis for the different outcomes between DSB induction and replication inhibition, we examined the activation of checkpoint kinases. We used activation-specific antibodies for checkpoint kinases: phosphorylated ATM (Ser1981), ATR (Thr1989) and Chk1 (Ser317 and 345) (Figure 3B) (60). (We could not determine an appropriate antibody for hamster Chk2 kinase.) Although both ATM and ATR (and downstream Chk1) were activated efficiently in both cell types by hydroxyurea and CPT treatment, such activation was missing in Mre11-KD cells in response to etoposide treatment. Expression of human MRE11 in Mre11-KD cells restored the activation of ATM and ATR. These results indicate that Mre11 is required for ATM/ATR-mediated cell cycle arrest in response to DSBs but is not required for the arrest in response to replication inhibition.

### ATM/ATR-mediated checkpoint suppresses gene amplification

In the previous sections, we showed that Mre11-KD cells have (1) increased DNA breaks during replication and (2) impaired checkpoint response to DSBs. Because DSBs promote gene amplification proficiency, the gene amplification proficiency in Mre11-KD cells could be solely due to the increase in DSBs. To determine whether this checkpoint defect itself could contribute to gene amplification proficiency, we measured gene amplification frequency in cells with either intact or impaired checkpoint using our I-SceI DSB-induced *DHFR* amplification system in CHO-dhfr- cells (D79IRSce) (8). In this system, an I-SceI-mediated chromosome break is induced next to a short (79 bp) inverted repeat sequence and promotes palindromic gene amplification of the *DHFR* transgene.

**Figure 4.**
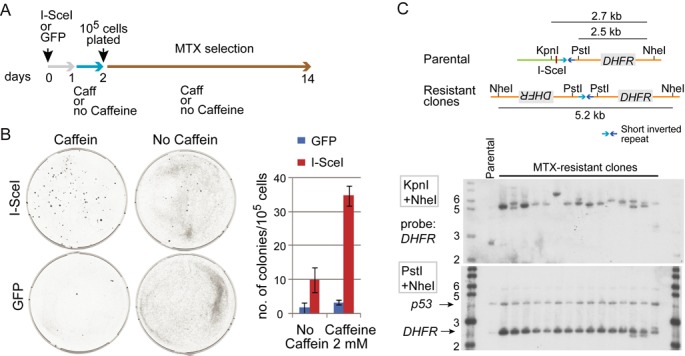
ATM/ATR checkpoint independently suppresses gene amplification proficiency. (**A**) Experimental design. Cells with wild-type Mre11 were transiently transfected with either I-SceI endonuclease expression vector (inducing a site-specific chromosomal DSB) or a control, GFP expressing vector. 10^5^ cells were plates and selected with MTX for 12 days. (**B**) MTX-resistant colonies arise more frequently cells with checkpoint abrogated by the treatment with caffeine. *Left*: representative plates from each experimental setting. *Right*: the average number of MTX-resistant colonies from three independent experiments. (**C**) Palindromic gene amplification in cells resistant to MTX. *Top:* double digest of DNA with *KpnI* and *NheI* confirmed that all the resistant cells underwent palindromic *DHFR* duplication. Briefly, the *KpnI* site was located distal to the *I-SceI* site and, if a DSB occurred at the *I-SceI* site and induces palindromic duplication, the *KpnI* site would be lost and the *NheI* fragment would be detected by a probe (*DHFR*). Consistent with palindromic duplication, all the resistant cells exhibited fragments of roughly 5 kb in size. *Bottom*: double digest of DNA with *PstI* and *NheI* confirmed *DHFR* amplification. This double digest yielded a 2.5 kb fragment from both parental and resistant cells, and the intensity of the fragment represented the copy number of *DHFR*. In all resistant cells, the intensities were much higher than that of parental cells (designated as P).

An I-SceI expression vector was transiently transfected into D79IRSce cells and MTX selection was conducted either in the presence or absence of caffeine (Figure 4A). Consistent with previous results, DSBs promoted gene amplification: cells transfected with an I-SceI expression vector exhibited significant increase of MTX-resistant colonies compared to cells transfected with a GFP expression vector. When cells with DSBs were selected by MTX in the presence of caffeine, colonies increased further (3–4-fold) (Figure 4B) (Supplementary Table S3). Southern blotting and restriction mapping was conducted for DNA from MTX-resistant cells (colonies), and we confirmed that MTX resistance of caffeine-treated cells was due to palindromic gene amplification (Figure 4C). These results indicate that an impaired ATM/ATR-mediated checkpoint can independently promote palindromic gene amplification.

### Global transcriptional changes associated with Mre11 deficiency

When we first introduced *Mre11* shRNA into cells, we observed growth arrest of the cell population; however, after several days in culture, cells resumed growth at a decreased rate (Figure 1B). Since Mre11 is essential for cell survival, some compensatory adaptive processes could take place and enabled cells to proliferate. To determine molecular signatures behind these changes, we performed global gene expression analysis using RNA-seq technology (Table 1, Supplementary Tables S4 and S5). First, we confirmed the significantly reduced mRNA abundance of *Mre11* (1714 and 2041 reads for control cells versus 632 and 721 reads for Mre11-KD cells, *P* < 10^−5^). Using Database for Annotation, Visualization and Integrated Discovery, we examined functional annotation of genes that showed significant differential expression between control and Mre11-KD cells (*P* < 0.005, Table 1A; Supplementary Table S4). The most significantly downregulated category (protein complex assembly) included genes that promote several cellular processes: *TUBA3A, TUBB2C, TUBA4A, TUBG1, TUBA1B, TUBB3* and *TUBA1C* (microtubule assembly), *PTRF* (transcription of ribosomal RNA genes), *ELF6* (translation initiation factor), *TTF2* (transcription termination and pre-mRNA splicing), *IPO5* and *NPM1* (trafficking between nucleus and cytoplasm), *FBXO5* (ubiquitin protein ligase complex). We also noted that many genes involved in cell cycle progression, DNA replication and repair were downregulated. Such genes included *Cyclin E1, Rad18, CDC6, Cdt1, Exo1* and *Ligase IV* (Table 1B). Global downregulation of these genes may indicate an alternative adaptive way for a cell with defective intra-S phase checkpoint to slow down replication in the presence of broken forks, which would provide more time for resuming replication (see the discussion).

**Table 1.** Differentially Expressed Genes

A					B		
Category	GO ID	GO term	Fold enrichment	*P*-value	Genes	Fold change (Mre11-KD/Con)	*P*-value
Mre11-knockdown > Control					Exo1	0.5	4.71E-17
Cholesterol biosynthesis	GO:0016126	sterol biosynthetic process	16.3	5.30E-13	RCC1	0.56	1.40E-06
	GO:0006695	cholesterol biosynthetic process	18.22	8.88E-12	LIG4	0.67	9.01E-06
Inflammatory response	GO:0006954	inflammatory response	3.1	2.49E-05	UBE2C	0.59	1.18E-05
	GO:0009611	response to wounding	2.41	1.62E-04	NTBP	0.64	2.14E-05
Regulation of cell proliferation	GO:0008285	negative regulation of cell proliferation	2.8	2.46E-04	EID1	0.74	4.39E-05
	GO:0042127	regulation of cell proliferation	1.94	7.92E-04	CCNE1	0.5	0.0003
					CDC6	0.45	0.0004
Control > Mre11-knockdown					RAN	0.64	0.0005
Protein Polymerization	GO:0051258	protein polymerization	14.53	1.02E-06	BIRC5	0.64	0.0008
	GO:0043623	cellular protein complex assembly	6.73	1.70E-05	CDT1	0.52	0.0013
Cell Cycle, DNA metabolism	GO:0007049	cell cycle	2.85	1.03E-05	PIN1	0.72	0.0019
Protein Transport	GO:0006913	nucleocytoplasmic transport	6.06	3.44E-04	MCM3	0.6	0.0022
	GO:0051169	nuclear transport	5.93	3.90E-04	NPM1	0.68	0.0023

A. Gene ontology analysis for differentially expressed genes between Mre11-KD cells and control cells. Three hundred eighty-nine upregulated genes and 187 downregulated genes were analyzed. Top three categories based on the significance value with GO ID and enrichment scores (enrichment) were listed. Enrichment was calculated as the number of genes that were called in the list/list total (either 389 or 187) divided by the total number of genes in the GO category/total number of genes annotated in the genome (13 588).

B. Genes in the cell cycle and DNA metabolism category that are significantly downregulated in Mre11-KD cells. Both fold increase and significance value are shown.

We found that genes involved in steroid biosynthesis and inflammation comprised two clusters that were most significantly upregulated in Mre11-KD cells. We found that several enzymes in this pathway including *HMGCS1, MVD, IDI1, FDPS, FDFT1, CYP51, SC4MOL, NSDHL* were simultaneously upregulated. This upregulation is notable, given the fact that Mre11-KD cells proliferate slowly and thus have presumably less demand for steroids for cellular structural constituents. The immune response group included genes involved in stimulation of proliferation leukemia inhibitory factor (LIF) and NF-kappaB and MAP kinase activation (*SQSTM1, TNFSF15*), various complement components, as well as genes involved in detection of abnormal and foreign molecules, including DNA in the cytoplasm (*TMEM173* (STING)). The genes involved in recognition of abnormal molecules could have a causative role in stimulation of the immune system, because, under replication stress, the cell may be overloaded with resected pieces of DNA. Such DNA ‘leakage’ to the cytoplasm may result in activating immune response through the STING recognition pathway (61).

## DISCUSSION

Gene amplification underlies aggressive tumor phenotypes by promoting cancer progression and therapy resistance. Despite the clinical importance, it is still unclear what renders cells proficient for gene amplification. We addressed the issue by creating genetically altered cells (Mre11-KD cells) that suffer from severe replication stress.

### The MRN complex prevents replication-associated DSBs

Mre11-KD cells experienced the increase of both ssDNA and dsDNA breaks during S-phase, indicating that the MRN complex prevents replication-associated breaks. This is consistent with the previous observation using *Xenopus* extracts (32,33), and explains why *Mre11*-null cells cause severe growth defects in higher eukaryotes (29–31). In yeast, *Mre11*-null strains are viable; however, growth defects were noted (62). Therefore, an evolutionary conserved role of the MRN complex is to ensure successful duplication of the genome by preventing spontaneous (endogenous) DSBs under normal growth conditions.

**Figure 5.**
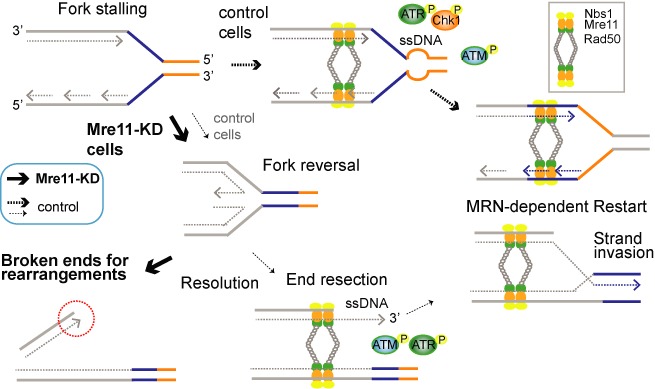
Model: the source of DNA breaks in Mre11-KD cells and mechanism of the MRN-dependent fork restart. Fork stalling, reversal and resolution results in one-ended DSBs for a substrate of rearrangement and gene amplification (thick solid line). In control cells with a sufficient MRN complex, fork restart is facilitated before fork reversal (thick dotted lines). Restart from one-ended breaks can also be facilitated by MRN-dependent activation of ATM/ATR and end resection (thin dotted line).

How do endogenous breaks arise during replication when MRN function is impaired? Our results collectively revealed that replication fork collapsing is a source of breaks in Mre11-KD cells: (i) SSBs precede DSBs (Figure 2C and D), (ii) DSBs are increased by checkpoint abrogation (Supplementary Figure S5) and (iii) Holliday junction-like structures, inferred by the sensitivity to a peptide WRWYCR, are more abundant in Mre11-KD cells (Figure 2E). Therefore, during replication, Mre11-KD cells likely experience massive fork reversals leading to fork collapses (Figure 5). The prevention of fork reversal by Mre11 has been noted previously in yeast when replication forks encountering an HO endonuclease-induced DSB (terminal forks) were examined (63). Two possible scenarios explain how the MRN complex prevents fork reversal in Mre11-KD cells. First, the MRN complex can play an active (structural) role by tethering sister chromatids together and preventing branch migration that leads to fork reversal. Second, four-way junctions can arise as a passive outcome of the failure to fork restart.

### Checkpoint defects in Mre11-KD cells

Mre11-KD cells were defective in executing checkpoint functions when cells to DSBs. Incomplete cell cycle arrest by etoposide was associated with the lack of the activation of ATM and ATR (Figure 3). This is consistent with the fact that the MRN complex is needed to activate ATM (22,23). The lack of ATR phosphorylation after etoposide treatment may indicate that processing of DSB ends into ssDNA was also compromised in Mre11-KD cells due to the lack of end resection (nuclease activity of Mre11). In contrast, we found a comparable level of both cell cycle arrest and ATR-Chk1 activation between control and Mre11-KD cells in response to replication inhibitors. Therefore, checkpoint for replication-associated ssDNA lesions was maintained in Mre11-KD cells.

In endogenous conditions, the uncoupling of replicative helicases and polymerases at stalled forks could lead to the accumulation of ssDNA and the subsequent activation of ATR and Chk1 (64,65). This activation can stabilize stalled forks and prevent fork collapse. In this regard, it is noteworthy that in Mre11-KD cells, despite having an activation of ATR-Chk1 to ssDNA, the increase in ssDNA lesions in S-phase (Figure 2D) was followed by a dramatic increase in DSBs. Therefore, in addition to the ATR-Chk1 checkpoint, the MRN complex *per se* is needed to protect stalled forks from collapsing in mammalian cells, as recently reported for yeast (66,67).

Taken together, the MRN complex is needed to suppress replication-associated DSBs, most likely by preventing fork reversal and facilitating the restart of stalled replication forks (Figure 5, top). One-ended replication-associated DSBs could offer another opportunity for the MRN complex to execute fork restart (68,69) (Figure 5, bottom). Such restart could require all three functions of the MRN complex: signaling function through ATM, structural function for holding sister chromatids together and nuclease function for processing one-ended DSBs. These processes ensure effective strand invasion into the sister chromatid for the reestablishment of the replication fork.

Finally, the fact that Mre11-KD cells could still proliferate with broken forks also suggests that there might be an alternative, MRN-independent fork restart mechanism from broken forks (63), although we cannot rule out the possibility that a residual amount of MRN activity in Mre11-KD cells was sufficient for the restart.

### Replication fork restart and cell viability in Mre11-KD cells

Our transcriptome analysis revealed that genes involved in DNA metabolism and replication were significantly downregulated in Mre11-KD cells. Among the genes, Exo1 is the most significantly downregulated gene (*P* < 10^−16^). Exo1 is a 5′ to 3′ exonuclease that co-operate with MRN-CtIP for the end resection of DSBs. Exo1 generates a long stretch of ssDNA that recruits ssDNA binding protein Replication Protain A (RPA) for checkpoint activation, strand invasion and HD repair (70,71). At stalled replication forks, Exo1 prevents fork reversal (in *Saccharomyces cerevisiae*) and promote Rad52 loading for recombinational restart (in *Schizosaccharomyces pombe*) (54,72). Thus, downregulation of *Exo1* could underlie the increased fork reversal and broken forks in Mre11-KD cells.

The significance of fork restart in eukaryotes remains unclear, because eukaryotic genomes carry multiple replication origins and converging forks could complete DNA replication when replication forks stall. However, the fact that genes in two replication fork protection systems, ATR (73) and Mre11, are required for cell viability indicates the essential role of fork restart in completing genome duplication in mammalian cells. In Mre11-KD cells, the transcription of genes that promote replication initiation (74,75), including *CDC6* (*P* = 0.0001), *Cdt1* (*P* = 0.001) and *MCM3* (*P* = 0.001), were significantly downregulated (Table 1B). Thus, promoting replication initiation unlikely rescues broken forks in Mre11-KD cells, but an alternative, Mre11-independent fork restart could sustain the viability.

### Mechanism of gene amplification in Mre11-deficient cells

In addition to the increase in DSBs and checkpoint defects, the observed intra-chromosomal amplification of the *DHFR* genes may indicate the previously defined role of Mre11 in suppressing palindromic gene amplification. Intra-chromosomal amplification can arise by several mechanisms, including limited re-replication of genomic segments, double rolling circle replication and Breakage-Fusion-Bridge (BFB) cycles (8,76–79). Among these mechanisms, BFB cycles involve hairpin-capped chromosomes as intermediates that would replicate to form inverted duplications of chromosomal regions. Hairpin-capped ends are preferred substrates of the endonuclease activity conferred by the MRX complex (and CtIP/Sae2 nuclease) in yeast (78,80–81). Hairpin-capped ends are created when DSBs were processed for a short distance by 5′ to 3′ resection, followed by fold-back annealing of 3′ tails to the same strand by microhomology (81). Such ends replicate to form inverted duplications in yeast lacking either *RAD50* or *Sae2* but not in wild-type cells. Therefore, one-ended DSBs from collapsed forks in Mre11-KD cells could be processed in a similar way to form hairpin-capped ends that lead to palindromic gene amplification. It is important to mention that such inverted duplications (fusions) can also be initiated from restarted forks, as recently shown in yeast (82).

## ACCESSION NUMBERS

GEO accession number for the RNA-seq data is GSE59487.

## SUPPLEMENTARY DATA

Supplementary Data are available at NAR Online.

SUPPLEMENTARY DATA
