# Supplementary material for: Replication fork integrity and intra-S phase checkpoint suppress gene amplification
Source: Nucleic Acids Res. 2015 Feb 11;43(5):2678–90. doi: 10.1093/nar/gkv084 (PMC4357702; doi:10.1093/nar/gkv084)
Supplement: SUPPLEMENTARY DATA [file supp_43_5_2678__index.html]

Replication fork integrity and intra-S phase checkpoint suppress gene amplification — SUPPLEMENTARY DATA 

# Replication fork integrity and intra-S phase checkpoint suppress gene amplification

## SUPPLEMENTARY DATA

**Files in this Data Supplement:**

- SUPPLEMENTARY DATA
