# Supplementary material for: Replication fork integrity and intra-S phase checkpoint suppress gene amplification
Source: Nucleic Acids Res. 2015 Feb 11;43(5):2678–90. doi: 10.1093/nar/gkv084 (PMC4357702; doi:10.1093/nar/gkv084)
Supplement: SUPPLEMENTARY DATA [file supp_gkv084_nar-03028-d-2014-File008.pdf]

Kondratova et al., - Replication fork protection and intra-S phase checkpoint suppress gene amplification

Supplementary Materials Table of Contents

Supplementary Methods

Supplementary figure 1 – Ongoing chromosome instability in Mre11-KD cells

Supplementary figure 2 – Intrachromosomal *DHFR* amplification (Fluorescence *in situ* hybridization)

Supplementary figure 3 – Tetraploidization does not precede gene amplification (chromosome count)

Supplementary figure 4 – Endogenous DNA breaks during replication (Comet assay)

Supplementary figure 5 – Checkpoint abrogation increases broken DNA in Mre11-KD cells (Pulse-Field Gel Electrophoresis)

Supplementary table 1 – The number of MTX-resistant colonies from 5 genotypes

Supplementary table 2 – The number of MTX-resistant colonies from fluctuation tests

Supplementary table 3 – The number of I-SceI induced MTX-resistant colonies

Supplementary table 4 – Gene ontology analysis for the differentially expressed genes (with gene lists)

Supplementary table 5 – The list of differentially expressed genes between Mre11-KD cells and control cells.

Supplementary methods

Growth curves and drug sensitivity

To obtain growth curves, $1x10^5$ cells were plated in triplicates for each time point in 6-well plates; each day cells were trypsinized and counted in triplicates. For drug sensitivity assays, cells were plated in triplicates in 96-well plates ($2x10^3$ cells/well) and were treated with serial dilutions of either etoposide (starting concentration 5uM) or hydroxyurea (starting concentration 500uM). For sensitivity of cells to Holliday Junction-binding peptide wrwycr, cells were treated with serial dilutions of the peptide (starting concentration 40 mkM) for 24h or 72h; cells treated for 24h were allowed to grow for additional 48h in drug-free medium before analysis. Cell viability was assessed using CellTiter-Blue® Cell Viability Assay (Promega).

Nuclear morphology

Nuclear morphology was studied in cells that were fixed with 4% formaldehyde and were stained with 100µg/ml DAPI (Molecular Probes, #D1306). Nuclei were observed by the fluorescent microscope equipped with digital camera; pictures were taken at either 20x, 40x or 63x magnification using Q-Capture Pro7 software. Nuclear sizes were analyzed using ImageJ software. For each DAPI-stained nucleus, integrated intensity of fluorescence was measured.

DHFR copy number analyses

DHFR copy number was measured using quantitative real time PCR using Light Cycler 480 (Roche, Indianapolis, IN, USA). Genomic DNA was extracted from cells as described

previously. Briefly, cells were incubated in the lysis buffer (100m*M* NaCl/10m*M* Tris·HCl, pH 8.0/25m*M* EDTA/0.5%SDS/proteinase K) for 24 hours at 37°C, followed by phenol/chloroform extraction and ethanol precipitation. PCR primers were designed for *DHFR* cDNA in the transgene and copy numbers were normalized to the internal control Chinese hamster *p53*. PCR reactions were carried out in a three-step 40-cycle reaction of 95°C for 30 seconds, 60°C for 3 seconds, and 72°C for 30 seconds by using iQ SYBR Green Supermix (Bio-Rad, Hercules, CA, USA). We used 5 ng/μl of genomic DNA for each reaction.

Southern blotting was done as described previously (references 8,9). 2μg of genomic DNA was digested with *PstI* and *NheI*, run on a 0.8% agarose gel and transferred onto nylon membrane. Probes (*DHFR* cDNA and Chinese hamster *p53*) were labeled with ^{32}P and co-hybridized to the membranes.

Supplementary figure 1 - Ongoing chromosome instability in Mre11-KD cells

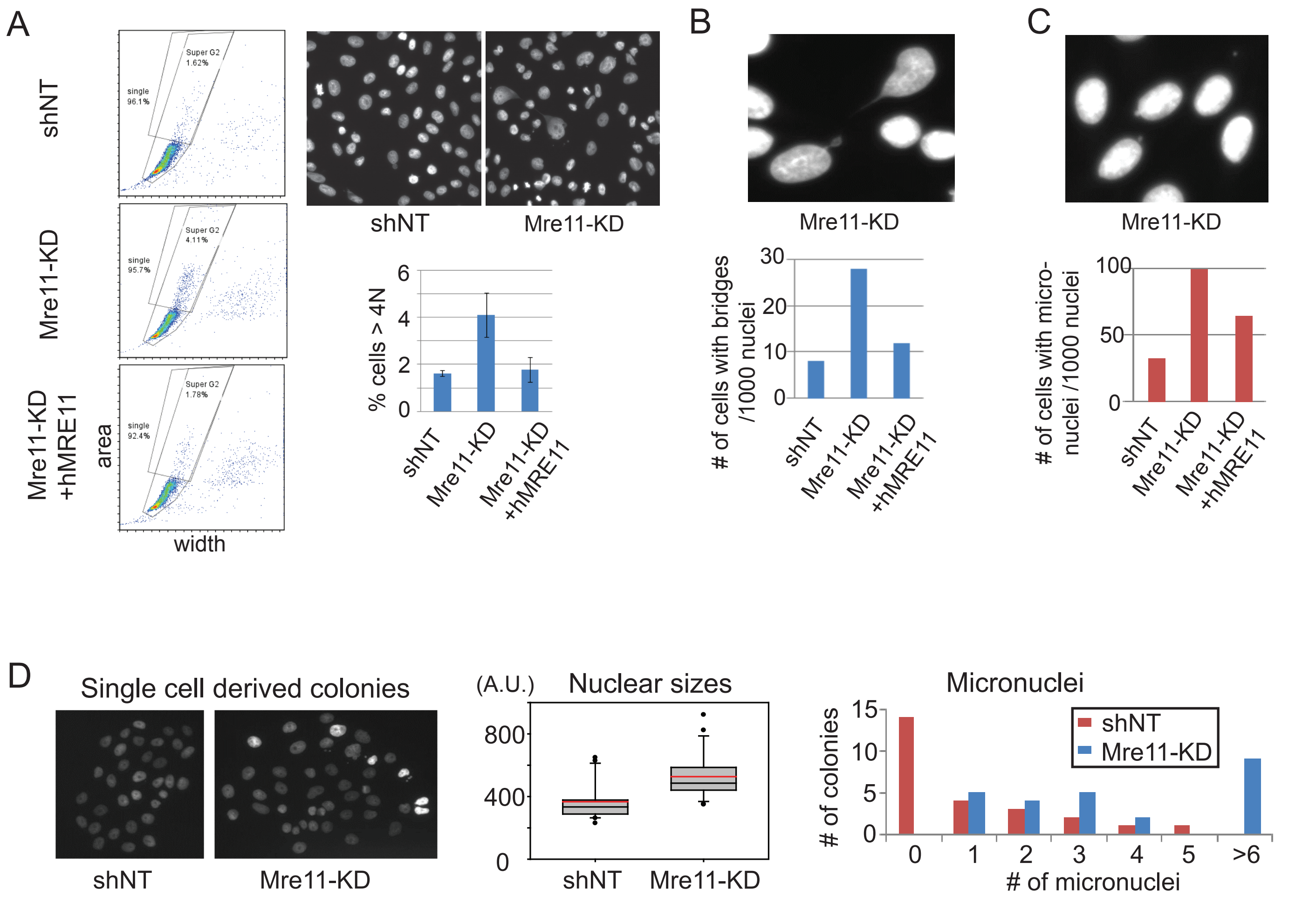

A. Cells with >4N DNA contents were increased in Mre11-KD cells. FACS profiles (left), nuclear morphology (nuclei stained with DAPI) (top) and a histogram for the fractions of cells with >4N DNA contents are shown. Sub-G1 cells would accumulate at the left-bottom corner of the diagrams.

B and **C**.The number of nuclei with chromatin bridges (E) and micronuclei (F) / 1000 nuclei. Nuclei stained with DAPI in Mre11-KD cells are shown on the top.

D. Ongoing chromosome instability in Mre11-KD cells. Left, colonies derived from single cells were isolated, fixed and stained with DAPI. Middle, average nuclear size (measured as integrated intensity of DAPI staining) within each colony. Right, for each colony, the number of micronuclei was counted.

Supplementary figure 2 - Intrachromosomal DHFR amplification

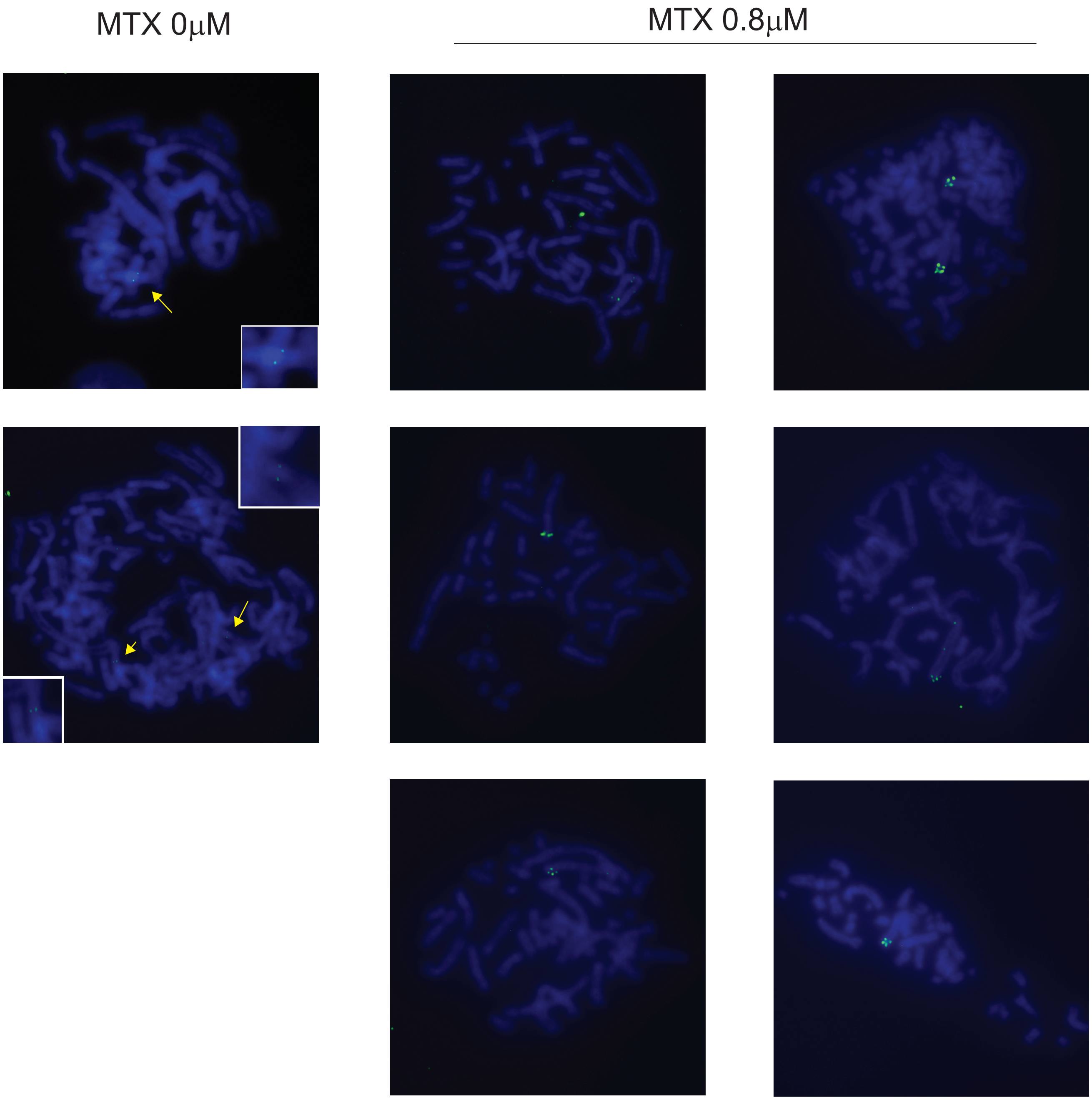

Fluorescense *in situ* hybridization of parental (left) and MTX-resistant (right) Mre11-KD cells. Note that both the numbers and fluorescense signal intensities are higher in MTX-resistant cells than parental cells.

Supplementary figure 3 – Chromosome count

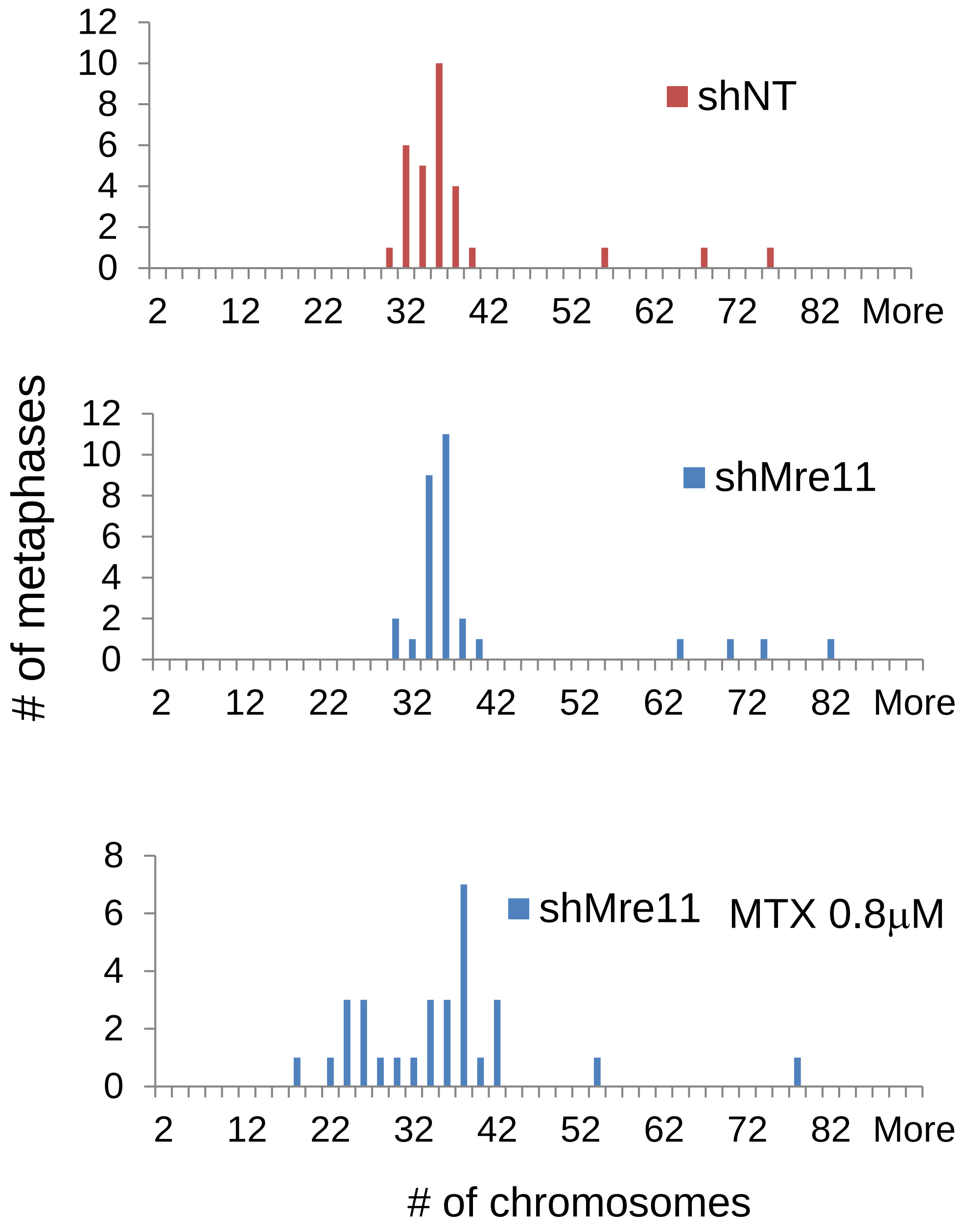

Tetraploidy unlikely precedes gene amplification. The number of chromosomes were counted from each genotype (30 metapases each) and are shown in the histograms. Note that chromosome loss, but not gain was evident in MTX-resistant cells.

Supplementary figure 4 - Endogenous DNA breaks during replication

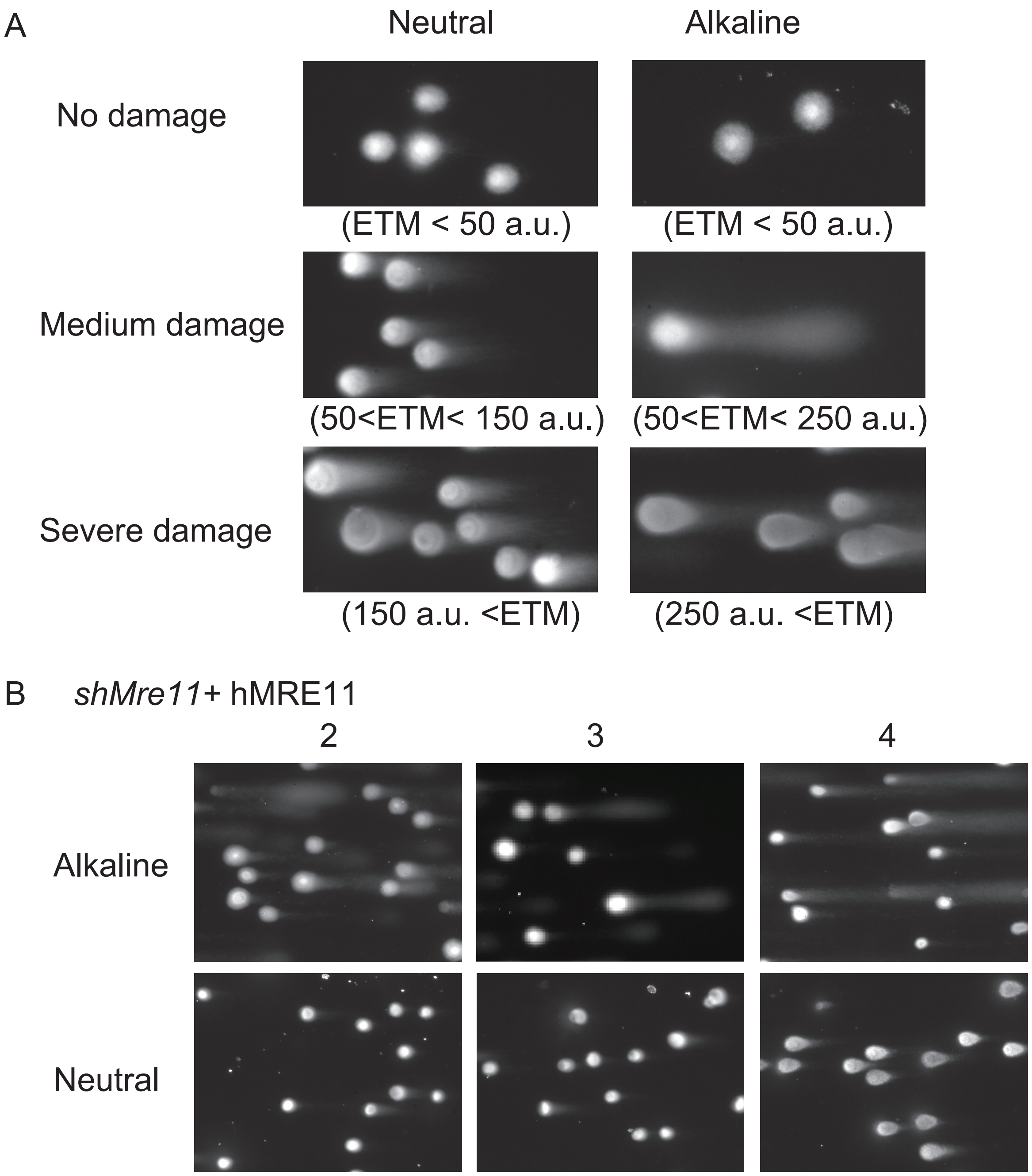

A. Representative images from comet assays performed in neutral and alkaline conditions are shown, demonstrating no damage, medium damage and severe damage.
B. Comet assay for Mre11-KD cells suplemented with human Mre11. Experimental conditions (2, 3 and 4) are described in Fig. 2C.

Supplementary figure 5 - Checkpoint abrogation increases broken DNA in Mre11-KD cells

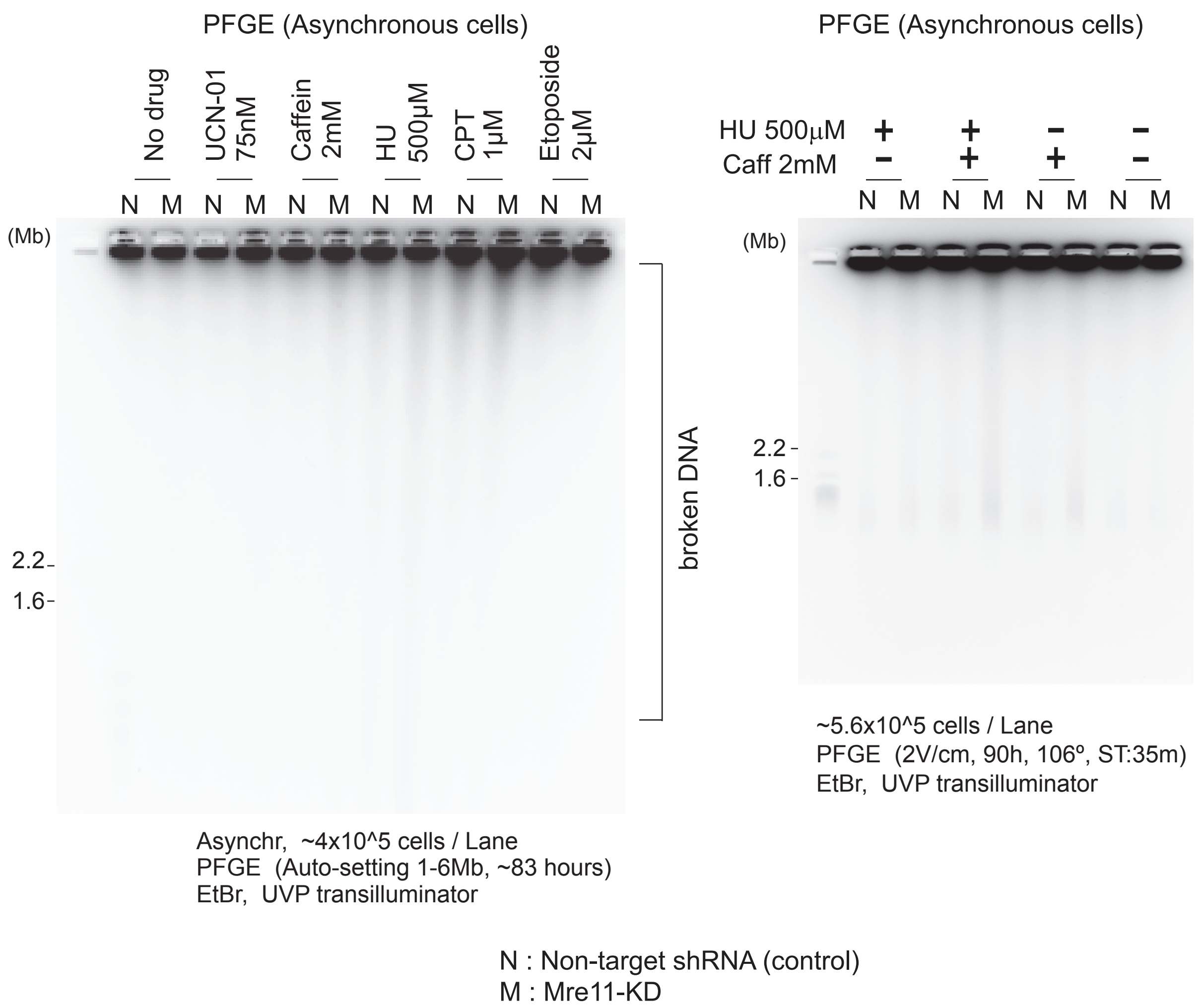

Checkpoint abrogation increased broken DNA. Cells were treated as indicated for 24 hours and were mounted into agarose blocks. Pulse field gel electrophoresis was performed to separate highmolecular weight DNA. Note that Caffein alone (6th lane from left in both the left and right panel) increases broken DNA in Mre11-KD cells cells more noticably than in control cells.

Supplementary table 1 The number of MTX resistant colonies from 5 genotypes (Fig. 1D)

Genotype			colony counts					relative # of colonies	
	MTX(μM)	# of cells plated	**Experiment 1**	**Experiment 2**	**Experiment 3**	**Average**	**STDEV**	**Average**	**STDEV**
**LV-GFP**	0.4	10,000	2	5	3	3.33	1.53	1.00	0.46
	0.8	100,000	2	4	3	3.00	1.00	1.00	0.33
shNT	0.4	10,000	15	11	6	10.67	4.51	3.20	1.35
	0.8	100,000	7	7	3	5.67	2.31	1.89	0.77
shMre11	0.4	10,000	58	50	53	53.67	4.04	16.10	1.21
	0.8	100,000	34	43	46	41.00	6.24	13.67	2.08
shMre11 **→ hMre11**	0.4	10,000	21	26	34	27.00	6.56	8.10	1.97
	0.8	100,000	19	14	13	15.33	3.21	5.11	1.07
**hMre11 →** ***shMre11***	0.4	10,000	10	8	7	8.33	1.53	2.50	0.46
	0.8	100,000	7	3	1	3.67	3.06	1.22	1.02

STDEV, standard deviation

Experimental procedure

Either 10^5 (for 0.8μM MTX) or 10^4 cells (for 0.4 μM MTX) were plated into a $10cm^2$ plate and, on the next day MTX was added to the media. Media and MTX were replaced every four days. After 12 days of selection, colonies on the plates were fixed and stained.

Supplementary table 2 Fluctuation test (MTX 0.8μM) (Fig. 1G)

clones	# of colonies / 10^5 cells
shNT-1	0
shNT-2	0
shNT-3	0
shNT-4	1
shNT-5	0
shNT-6	0
shNT-7	0
shNT-8	0
shNT-9	1
shNT-10	6
Mre11-KD-1	3
Mre11-KD-2	37
Mre11-KD-3	15
Mre11-KD-4	10
Mre11-KD-5	0
Mre11-KD-6	0
Mre11-KD-7	0
Mre11-KD-8	0
Mre11-KD-9	0
Mre11-KD-10	0

Experimental procedure

10 single-cell derived cell clones were isolated for both control (*shNT*) and Mre11-KD cells. Clones were expanded up to 10^6 cells and 10^5 cells were plated onto a 10 cm^2 plate for MTX selection (0.8μM). After 12 days, plates were stained with 0.1% crystal violet solution and colonies were counted.

Supplementary table 3

Expression vector	Colony count with caffeine	without caffeine
Sce-1	32	7
Sce-2	34	9
Sce-3	38	14
GFP-1	3	0
GFP-2	3	2
GFP-3	4	3

Experimental procedure

1. transfection
Either 5 μg of I-SceI or GFP expression vector was transfected to $3x10^5$ D79-8 cells.
After 3 hours of transfection, $1x10^5$ cells were plated into six 10 cm plate.

2. Caffeine treatment
The following day, caffeine was added into the three of 6 plates.

3. MTX and Caffeine treatments
The following day, media was replaced with the one with 0.4μM MTX. Caffein was added to the three plates. The treatment was repeated every four days.

4. After 12 days of MTX selection, plates were stained with crystal violet.

Supplementary table 4

GO ID	GO cluster	PValue	Genes
**Genes downregulated in Mre11-KD cells**			
**1 Protein Polymerization**			
GO:0051258	protein polymerization	1.02E-06	TUBA3A, TUBB2C, FBXO5, TUBA4A, TUBG1, TUBA1B, TUBB3, TUBA1C
GO:0043623	cellular protein complex assembly	1.70E-05	TUBA3A, IPO5, TUBB2C, FBXO5, TUBA4A, TUBG1, KPNB1, TUBA1B, TUBB3, TUBA1C
GO:0034621	cellular macromolecular complex subunit organization	3.27E-05	EIF6, TUBB2C, TUBA3A, TTF2, PTRF, IPO5, H2AFZ, TUBA4A, FBXO5, TUBG1, KPNB1, TUBA1B, TUBB3, TUBA1C
GO:0070271	protein complex biogenesis	6.81E-05	TUBB2C, TUBA3A, PTRF, NPM1, IPO5, C1QTNF2, TUBA4A, FBXO5, TUBG1, KPNB1, TUBA1B, TUBA1C, TUBB3
GO:0006461	protein complex assembly	6.82E-05	TUBB2C, TUBA3A, PTRF, NPM1, IPO5, C1QTNF2, TUBA4A, FBXO5, TUBG1, KPNB1, TUBA1B, TUBA1C, TUBB3
GO:0043933	macromolecular complex subunit organization	1.60E-04	EIF6, TUBA3A, TUBB2C, TTF2, PTRF, C1QTNF2, NPM1, IPO5, H2AFZ, TUBA4A, FBXO5, TUBG1, KPNB1, TUBA1B, TUBB3, TUBA1C
GO:0034622	cellular macromolecular complex assembly	1.99E-04	EIF6, TUBA3A, IPO5, TUBB2C, H2AFZ, FBXO5, TUBA4A, TUBG1, KPNB1, TUBA1B, TUBB3, TUBA1C
GO:0065003	macromolecular complex assembly	2.32E-04	EIF6, TUBB2C, TUBA3A, PTRF, C1QTNF2, NPM1, IPO5, H2AFZ, TUBA4A, FBXO5, TUBG1, KPNB1, TUBA1B, TUBB3, TUBA1C
GO:0007017	microtubule-based process	6.55E-04	TUBA3A, TUBB2C, NPM1, FBXO5, TUBA4A, RANBP1, BIRC5, TUBG1, TUBA1B, TUBB3, TUBA1C
**2 Cell Cycle, DNA metabolism**			
GO:0007049	cell cycle	1.03E-05	EXO1, EID1, CDC6, RAN, MRE11A, BIRC5, LIG4, PMF1, MCM3, RCC1, UBE2C, PIN1, CDT1, CCNE1, NPM1, MTBP, FBXO5, RANBP1, TUBG1, RUVBL1, LFNG, TUBB3, TXNL4A, BLCAP
GO:0000279	M phase	3.52E-05	EXO1, CDC6, RAN, MRE11A, BIRC5, PMF1, RCC1, UBE2C, FBXO5, RANBP1, RUVBL1, TUBG1, LFNG, TUBB3, TXNL4A
GO:0022403	cell cycle phase	4.56E-05	EXO1, CDC6, RAN, MRE11A, BIRC5, PMF1, RCC1, UBE2C, MTBP, FBXO5, RANBP1, RUVBL1, TUBG1, LFNG, TUBB3, TXNL4A
GO:0022402	cell cycle process	1.01E-04	EXO1, CDC6, RAN, MRE11A, BIRC5, PMF1, RCC1, UBE2C, NPM1, MTBP, FBXO5, RANBP1, RUVBL1, TUBG1, LFNG, TUBB3, TXNL4A
GO:0000278	mitotic cell cycle	5.35E-04	CDC6, RAN, MTBP, FBXO5, BIRC5, TUBG1, PMF1, RUVBL1, UBE2C, RCC1, TUBB3, TXNL4A
GO:0006259	DNA metabolic process	6.84E-04	HMGN1, EXO1, CDC6, HMGB2, MRE11A, HSPA1A, LIG4, RRM2B, MCM3, RMI1, CDT1, CCNE1, SFPQ, PCNA, RAD18, RUVBL1
GO:0051726	regulation of cell cycle	7.32E-04	CCNE1, HMGB1, JUN, NPM1, MTBP, FBXO5, RANBP1, BIRC5, TUBG1, HSPA8, CDT1
**3 Protein Transport**			
GO:0006913	nucleocytoplasmic transport	3.44E-04	NXT1, RAN, IPO5, NPM1, KPNA2, KPNB1, HNRNPA1, TOB1
GO:0051169	nuclear transport	3.90E-04	NXT1, RAN, IPO5, NPM1, KPNA2, KPNB1, HNRNPA1, TOB1
GO:0006605	protein targeting	4.84E-04	NXT1, YWHAG, RAN, TOMM5, IPO5, TIMM13, KPNA2, KPNB1, TOB1
GO:0017038	protein import	8.98E-04	RAN, TOMM5, IPO5, TIMM13, KPNA2, KPNB1, TOB1
GO:0031328	positive regulation of cellular biosynthetic process	5.76E-04	FUS, HMGB1, HMGB2, HSP90AA1, GLIS2, TLR2, ZEB1, SOX8, HMGA1, PPARGC1B, CBFB, GATA2, ETS1, JUN, C1QTNF2, NPM1, DYRK2, RBM14, EIF5A2
GO:0009891	positive regulation of biosynthetic process	6.40E-04	FUS, HMGB1, HMGB2, HSP90AA1, GLIS2, TLR2, ZEB1, SOX8, HMGA1, PPARGC1B, CBFB, GATA2, ETS1, JUN, C1QTNF2, NPM1, DYRK2, RBM14, EIF5A2
**Genes upregulated in Mre11-KD cells**			
**1 Cholesterol biosynthesis**			
GO:0016126	sterol biosynthetic process	5.30E-13	CYB5R3, CYP51, EBP, SC5D, MVD, HMGCS1, FDPS, FDFT1, SC4MOL, INSIG2, INSIG1, IDI1, NSDHL, DHCR24
GO:0006695	cholesterol biosynthetic process	8.88E-12	CYP51, CYB5R3, EBP, MVD, INSIG2, INSIG1, FDPS, HMGCS1, IDI1, NSDHL, DHCR24, FDFT1

Supplementary table 4

GO:0006694	steroid biosynthetic process	9.97E-12	CYB5R3, CYP51, HSD3B2, EBP, SC5D, MVD, HMGCS1, FDPS, LSS, FDFT1, SC4MOL, INSIG2, PXMP3, INSIG1, SRD5A1, IDI1, DHCR24, NSDHL
GO:0016125	sterol metabolic process	3.10E-08	CYB5R3, CYP51, EBP, SC5D, MVD, HMGCS1, FDPS, FDFT1, SC4MOL, SREBF2, INSIG2, INSIG1, IDI1, DHCR24, NSDHL
GO:0008203	cholesterol metabolic process	5.92E-07	CYB5R3, CYP51, EBP, MVD, HMGCS1, FDPS, FDFT1, SREBF2, INSIG2, INSIG1, IDI1, NSDHL, DHCR24
GO:0008610	lipid biosynthetic process	6.53E-07	CYB5R3, CYP51, HSD3B2, SC5D, PTGS2, MVD, HMGCS1, LSS, SC4MOL, FDFT1, INSIG2, ANG, PXMP3, INSIG1, PIGC, ELOVL7, SRD5A1, PPAP2A, NSDHL, DHCR24, EBP, FDPS, DHRS9, LPCAT3, DGAT2, IDI1
GO:0008202	steroid metabolic process	7.90E-07	HSD3B2, CYB5R3, CYP51, EBP, SC5D, MVD, HMGCS1, FDPS, LSS, FDFT1, SC4MOL, SREBF2, INSIG2, PXMP3, INSIG1, SRD5A1, IDI1, DHCR24, NSDHL
GO:0008299	isoprenoid biosynthetic process	3.26E-04	MVD, FDPS, HMGCS1, DHRS9, IDI1, FDFT1
GO:0006720	isoprenoid metabolic process	4.48E-04	RDH11, MVD, FDPS, HMGCS1, DHRS9, IDI1, BCO2, FDFT1
**2 inflamatory responses**			
GO:0006954	inflammatory response	2.49E-05	NFKBIZ, CCL2, C3, LY96, CXCL3, PPARG, TLR1, F8, IL25, SAA3, TLR3, C1RA, NOD2, NUPR1, CCL20, C1RL, SCN9A, CFH, VNN1, CR1L
GO:0009611	response to wounding	1.62E-04	NFKBIZ, CCL2, C3, LY96, CXCL3, PPARG, F2RL1, TLR1, NINJ2, F8, IL25, SAA3, GJA1, TLR3, C1RA, NOD2, NUPR1, CCL20, C1RL, SCN9A, CFH, VNN1, LCP1, CR1L
GO:0045087	innate immune response	2.37E-04	C1RA, TMEM173, NOD2, C3, CEBPG, TLR1, C1RL, CFH, TLR3, VNN1, PRDX1, CR1L
GO:0006955	immune response	4.72E-04	MFSD6, CCL2, C3, CXCL3, TLR1, TNFSF15, TLR3, ACP5, BNIP3, PRDX1, LIF, C1RA, TMEM173, NOD2, CCL20, SQSTM1, CFH, VNN1, H2-K1, LY96, CEBPG, ZFR2, BCAP31, LAT2, VAMP7, C1RL, LCP1, CR1L
**3 regulation of cell proliferation**			
GO:0008285	negative regulation of cell proliferation	2.46E-04	PTGS2, PPARG, NDFIP1, PAWR, SFN, CTNNA1, DDR1, SPRY2, CDKN1A, SPRY1, EREG, SERPINF1, NUPR1, CDKN2C, ANG, TGIF1, ATPIF1, TES
GO:0042127	regulation of cell proliferation	7.92E-04	SAT1, CCL2, PTGS2, IL6ST, PPARG, PAWR, SFN, LIF, SPRY2, SPRY1, CDKN2C, ANG, TGM2, FGFBP1, TES, IRS2, CRIP2, NDFIP1, ANXA1, CTNNA1, ATF5, DDR1, CDKN1A, EREG, SERPINF1, NUPR1, DBP, SERPINB5, TGIF1, ATPIF1

GO-ID and clusters in DAVID that are up- or down-regulated in Mre11-KD cells are listed along with the gene names in each GO cluster.

Supplementary table 5 differentially expressed genes

	Annotation	the Numbers of 35-bp reads				FDRsum	logPvaluesum
**gene symbol**		**control-1**	**control-2**	**Mre11KD-1**	**Mre11-KD-2**		
SLPI	antileukoproteinase	34	32	9980	8168	5.173E-191	1.7622E-195
FGFBP1	fibroblast growth factor-binding protein 1	0	0	1928	886	5.8064E-78	9.39618E-82
Sprr1a	cornifin-A	1217	1295	6334	8817	3.7036E-70	7.25503E-74
Prnp	major prion protein	701	722	3663	3257	1.6268E-65	4.01798E-69
BEX2	protein BEX2	197	245	1160	1162	1.3803E-63	3.98937E-67
Gcnt3	beta-1,3-galactosyl-O-glycosyl-glycoprotein beta-1,6-N-ac	2	2	210	164	2.8321E-59	9.40737E-63
Hnrnpd	heterogeneous nuclear ribonucleoprotein D0	2822	3468	222	320	1.026E-57	3.84476E-61
Sfn	14-3-3 protein sigma	6	8	324	565	3.6577E-45	2.21185E-48
Vasn	vasorin	1423	1684	542	662	8.1547E-34	1.13904E-36
Ier3	radiation-inducible immediate-early gene IEX-1	1902	1965	5567	6116	1.0437E-31	1.72448E-34
Jun	transcription factor AP-1	2206	2650	897	1015	2.9936E-31	5.20121E-34
Cxcl3	Cxcl3	750	956	3128	3644	2.4366E-29	4.83529E-32
Serpinb5	serine peptidase inhibitor, clade B, member 5	7	7	781	912	8.0717E-28	1.77368E-30
Vamp7	vesicle-associated membrane protein 8	870	1014	3265	3040	1.7542E-27	3.95919E-30
Sema4d	semaphorin 4D	4	5	70	74	3.7953E-27	8.82474E-30
F2rl1	coagulation factor II receptor 1, transcript variant 2	24	22	118	133	2.7247E-25	7.30695E-28
F2rl1	coagulation factor II %28thrombin%29 receptor 1, transcr	24	22	118	133	2.7247E-25	7.30999E-28
Gpx1	glutathione peroxidase 1	5660	6023	2467	2824	1.2945E-24	3.69018E-27
Blcap	bladder cancer-associated protein	1035	1135	487	505	7.638E-24	2.3354E-26
Fah	fumarylacetoacetase	434	447	3724	3230	2.4228E-23	7.63261E-26
Col8a1	collagen alpha-1%28VIII%29 chain	356	444	1766	2418	5.1886E-23	1.67926E-25
Id3	DNA-binding protein inhibitor ID-3	1704	2064	377	581	7.2463E-23	2.36377E-25
FHL1	four and a half LIM domains protein 1, transcript variant 1	1276	1484	123	194	2.7233E-22	9.26931E-25
FHL1	four and a half LIM domains protein 1, transcript variant 2	1276	1484	123	194	2.7233E-22	9.27762E-25
Foxs1	forkhead box protein S1	562	617	247	260	3.9216E-22	1.34938E-24
Klhl34	kelch-like protein 34	66	78	208	254	4.2578E-22	1.47592E-24
SOCS2	suppressor of cytokine signaling 2	208	232	719	645	6.5556E-21	2.52365E-23
Dag1	dystroglycan 1	12385	13840	25541	27034	4.4026E-20	1.8786E-22
Tmem125	transmembrane protein 125	1	0	44	40	5.8896E-20	2.54823E-22
Pla2g6	phospholipase A2, membrane associated	11	5	380	265	1.1702E-19	5.28219E-22
Plet1	placenta-expressed transcript 1 protein	451	489	1852	1672	4.5309E-19	2.10504E-21
Pex11a	peroxisomal membrane protein 11A	40	32	145	139	6.1811E-19	3.01652E-21
Dhrs9	dehydrogenase/reductase SDR family member 9	26	29	283	300	9.1545E-19	4.55113E-21
Bpgm	bisphosphoglycerate mutase	386	517	974	1155	3.3367E-18	1.80351E-20
Mvd	diphosphomevalonate decarboxylase	1957	1922	5488	5968	5.1326E-17	3.12852E-19
Gramd1c	GRAM domain-containing protein 1C	3	2	53	43	5.6456E-17	3.50046E-19
Tnfsf9	tumor necrosis factor ligand superfamily member 9	2627	2961	450	563	6.6887E-17	4.2042E-19
Tlr2	toll receptor 2	269	323	116	135	7.5911E-17	4.81023E-19
Chrnb1	cholinergic receptor, nicotinic, beta polypeptide 1	97	121	1173	1050	8.6375E-17	5.50273E-19
them6	UPF0670 protein C8orf55 homolog	407	382	1363	1354	8.983E-17	5.73813E-19
Foxa1	hepatocyte nuclear factor 3-alpha	16	17	76	83	2.3356E-16	1.55757E-18
Inhbb	inhibin beta B chain	785	989	357	471	2.9066E-16	1.95322E-18
SH3TC1	SH3 domain and tetratricopeptide repeats-containing prot	4	4	54	46	3.6719E-16	2.49875E-18
Tubb4a	tubulin, beta 4A class IVA	1548	1942	653	916	4.9295E-16	3.41338E-18
Vnn1	vanin 1	3796	4904	9015	9511	1.0147E-15	7.16559E-18
Gja1	gap junction protein, alpha 1	7243	8349	14276	13951	2.0808E-15	1.49721E-17
Trib3	tribbles homolog 3	779	852	3745	2632	2.9452E-15	2.13779E-17
Rnasel	ribonuclease 4 (2-5A-dependent ribonuclease)	136	171	588	437	4.3586E-15	3.29647E-17
Exo1	Exo1	854	1028	403	529	6.0522E-15	4.71137E-17
Ppap2a	lipid phosphate phosphohydrolase 1	903	1122	2400	2550	1.0948E-14	8.78177E-17
Tob1	protein Tob1	249	307	115	131	1.1405E-14	9.18939E-17
Krt8	keratin, type II cytoskeletal 8	0	2	29	38	1.7963E-14	1.48709E-16
Fibin	fin bud initiation factor homolog	3	4	80	44	2.2028E-14	1.85173E-16
Itm2b	integral membrane protein 2B	541	678	2317	1814	3.5214E-14	3.0632E-16
Tmem173	transmembrane protein 173	6	4	427	388	3.6258E-14	3.16835E-16
Ddit4	DNA damage-inducible transcript 4 protein	80	72	271	297	3.9836E-14	3.50482E-16
Tnfsf15	tumor necrosis factor ligand superfamily member 15	0	0	26	27	4.5178E-14	4.00176E-16
Cspg4	chondroitin sulfate proteoglycan 4	7367	8219	878	1552	5.9342E-14	5.36243E-16
Filip1l	filamin A interacting protein 1	170	179	587	415	6.2088E-14	5.63707E-16
Aatk	apoptosis-associated tyrosine kinase	9	2	48	51	6.9937E-14	6.37347E-16
Hpse	heparanase	494	599	2408	2317	1.0153E-13	9.65929E-16
Fdps	farnesyl pyrophosphate synthase	4263	4732	12962	14047	1.0496E-13	1.0012E-15
Atf4	Atf4	2179	2449	5576	4841	1.2221E-13	1.17723E-15
Tcf19	transcription factor 19-like protein	668	743	199	334	2.1758E-13	2.15664E-15
Thnsl1	threonine synthase-like 1	206	205	525	438	2.2124E-13	2.20273E-15
Itm2c	integral membrane protein 2C	5932	6538	14142	12743	3.5851E-13	3.56344E-15
Saa3	serum amyloid A-3 protein	0	0	308	134	5.4869E-13	5.77145E-15
Ngfrap1	protein BEX3	677	779	1385	1296	6.0986E-13	6.44075E-15

Mfsd6	major facilitator superfamily domain-containing protein 6	209	283	1668	1628	8.1754E-13	8.74549E-15
Arpc1b	actin-related protein 2/3 complex subunit 1B	2124	2247	3762	3973	1.9696E-12	2.20679E-14
Tspan7	tetraspanin-7	1223	1389	3574	3231	2.2795E-12	2.59952E-14
Pnldc1	poly(A)-specific ribonuclease PARN domain-containing pr	61	96	318	210	2.3216E-12	2.65944E-14
Ang	angiogenin	21	21	83	76	3.3268E-12	3.89318E-14
SLC39A2	zinc transporter ZIP2	3	3	42	33	5.0571E-12	6.09027E-14
Actg1	actin, cytoplasmic 2	56322	64882	32142	37602	7.2536E-12	8.84403E-14
Slco4a1	solute carrier organic anion transporter family, member 4a	2	0	26	28	7.2194E-12	8.94034E-14
Mgat2	alpha-1,6-mannosyl-glycoprotein 2-beta-N-acetylglucosar	1955	2263	1061	1338	7.6094E-12	9.46219E-14
Acta1	actin, alpha skeletal muscle A	2098	2500	1209	1477	8.1808E-12	1.02145E-13
Dnajb1	dnaJ homolog subfamily B member 1	1833	2070	728	987	1.0686E-11	1.35066E-13
Sc4mol	c-4 methylsterol oxidase	2893	3476	6876	6386	1.2587E-11	1.61307E-13
Phyhip	phytanoyl-CoA hydroxylase-interacting protein	1	2	29	28	1.3414E-11	1.72624E-13
Idh1	isocitrate dehydrogenase [NADP] cytoplasmic	2066	2537	5410	5127	1.3792E-11	1.7761E-13
Lss	lanosterol synthase	1978	1993	7585	8186	1.4154E-11	1.80225E-13
Reep5	receptor expression-enhancing protein 5	1002	1151	2482	2488	1.4657E-11	1.81861E-13
Tuba1b	tubulin alpha-1B chain	13741	15692	5970	8662	1.6009E-11	2.09562E-13
Gabarapl1	gamma-aminobutyric acid receptor-associated protein-like	1028	1185	3562	3134	1.9587E-11	2.60077E-13
Bnc2	basonuclin 2, transcript variant 2	29	36	4	2	2.4249E-11	3.28108E-13
Bnc2	basonuclin 2, transcript variant 1	29	36	4	2	2.4249E-11	3.28173E-13
Tuba1b	Tuba1b	64159	73791	26187	38340	2.6401E-11	3.42154E-13
Tpm1	tropomyosin alpha-1 chain, transcript variant 2	1786	2193	4917	4383	2.8384E-11	3.72675E-13
Tpm1	tropomyosin alpha-1 chain, transcript variant 6	853	1062	2295	2094	2.8384E-11	3.72809E-13
Pnrc2	proline-rich nuclear receptor coactivator 1	162	166	315	340	3.0005E-11	4.11697E-13
C1ra	calcium-dependent serine proteinase	2471	2749	8614	6911	3.2949E-11	4.52044E-13
Ddr1	discoidin domain receptor family, member 1, transcript va	974	967	3443	3446	3.7944E-11	5.22291E-13
Mex3b	RNA-binding protein MEX3B	132	134	54	49	4.4613E-11	6.28469E-13
Tuba4a	tubulin alpha-4A chain	6813	7791	2617	4062	6.1665E-11	8.55342E-13
TMEM217	transmembrane protein 217	7	4	62	39	7.6995E-11	1.12137E-12
Rdh11	retinol dehydrogenase 11	1000	1138	3188	3081	1.0181E-10	1.51995E-12
Il24	interleukin-24	0	0	20	21	1.0335E-10	1.54652E-12
Lamb3	laminin, beta 3	22	35	1529	1250	1.1113E-10	1.66223E-12
Tpk1	thiamin pyrophosphokinase 1	39	41	115	106	1.3405E-10	2.05158E-12
CXCL16	c-X-C motif chemokine 16	701	807	1739	1835	1.4792E-10	2.18877E-12
Atxn1	ataxin 1	28	34	136	117	1.6808E-10	2.51708E-12
Ckmt1	creatine kinase, mitochondrial 1, ubiquitous, transcript var	26	31	524	476	1.6351E-10	2.53714E-12
Ckmt1	creatine kinase, mitochondrial 1, ubiquitous, transcript var	26	32	530	478	1.6384E-10	2.5423E-12
Nupr1	nuclear protein 1	931	1105	2183	1911	1.8039E-10	2.67244E-12
Hspa8	heat shock cognate 71 kDa protein	40208	47225	19483	27310	1.8772E-10	2.97066E-12
Hoga1	4-hydroxy-2-oxoglutarate aldolase 1	43	51	123	117	2.3954E-10	3.82751E-12
Fst	follistatin	180	204	558	660	3.1823E-10	5.005E-12
Ptgs2	prostaglandin-endoperoxide synthase 2	306	379	1742	1867	4.0843E-10	6.79364E-12
Sqle	squalene monooxygenase	4159	4400	6831	7377	5.2683E-10	8.91882E-12
IFI27	interferon alpha-inducible protein 27, mitochondrial	14	6	104	72	5.9633E-10	9.81601E-12
Dtymk	thymidylate kinase	1883	2218	934	1206	6.5042E-10	1.06007E-11
Pkp3	plakophilin 3	694	784	2368	2712	7.5184E-10	1.2645E-11
Pirt	phosphoinositide-interacting protein	1263	1710	2330	2667	7.4048E-10	1.28467E-11
Slc23a3	solute carrier family 23, member 3, transcript variant 2	0	0	24	17	7.4671E-10	1.29676E-11
Slc23a3	solute carrier family 23 member 3, transcript variant 1	0	0	24	17	7.47E-10	1.29789E-11
Spsb2	SPRY domain-containing SOCS box protein 4	226	253	20	49	7.9582E-10	1.3922E-11
Nfil3	nuclear factor, interleukin 3, regulated	404	367	778	728	8.0063E-10	1.4013E-11
Epm2aip1	EPM2A-interacting protein 1	536	610	976	950	8.1192E-10	1.42244E-11
Tgm2	transglutaminase 2, C polypeptide	1179	1353	3853	4429	8.4612E-10	1.4832E-11
Gfod1	glucose-fructose oxidoreductase domain containing 1	153	183	397	407	9.5778E-10	1.69837E-11
Trp53inp1	tumor protein p53-inducible nuclear protein 2	16	13	104	83	9.9675E-10	1.7623E-11
Tuba3a	tubulin alpha-3 chain	21	24	1	1	1.2717E-09	2.31457E-11
Exd1	Exd1	90	120	253	210	1.285E-09	2.34205E-11
Hspa8	heat shock cognate 70 kDa protein	52715	62565	25771	36135	1.3711E-09	2.49545E-11
Tnrc18	zinc finger protein 469	887	885	490	543	1.3982E-09	2.56635E-11
AMAC1	protein AMAC1	14	9	48	51	2.0345E-09	3.84513E-11
Ascl2	achaete-scute homolog 2	270	232	61	87	2.5147E-09	4.85977E-11
Arpc1b	actin related protein complex, subunit 1B	11146	11883	19753	20773	2.7369E-09	4.86412E-11
HMGCS1	hydroxymethylglutaryl-CoA synthase, cytoplasmic	7961	8805	19496	23271	2.5666E-09	4.96431E-11
Lrrc8d	leucine-rich repeat-containing protein 8D	739	890	1482	1326	2.7092E-09	5.27018E-11
Tlr3	toll receptor 3	19	18	459	360	3.027E-09	5.92107E-11
Insig1	Insig1	1542	1666	3418	3846	3.3109E-09	6.37869E-11
Lgals3	galectin-3	6012	7228	11379	12440	3.4657E-09	6.5406E-11
Tmeff2	tomoregulin-2	55	51	1	3	3.3905E-09	6.72825E-11
Msn	moesin	9324	11408	3016	4016	3.5439E-09	7.06208E-11
Zbtb38	zinc finger and BTB domain-containing protein 38	467	540	993	842	3.9909E-09	8.0251E-11

Prdx1	Prdx1	9184	11522	17344	17514	6.3271E-09	1.31315E-10
Npr1	atrial natriuretic peptide receptor 1	3	5	37	28	8.6747E-09	1.85075E-10
CCL2	c-C motif chemokine 2	392	535	1035	939	1.0258E-08	2.21761E-10
Trp53inp1	tumor protein p53-inducible nuclear protein 1, transcript v	36	50	362	268	1.026E-08	2.22103E-10
Trp53inp1	tumor protein p53-inducible nuclear protein 1, transcript v	36	50	362	268	1.026E-08	2.22131E-10
Tmem65	transmembrane protein 65	553	792	313	409	1.1392E-08	2.48959E-10
Gsn	gelsolin	280	289	1589	1720	1.1609E-08	2.54181E-10
Cxcr7	chemokine %28C-X-C motif%29 receptor 7	1235	1664	494	617	1.2023E-08	2.64194E-10
Crtap	cartilage-associated protein	1858	2167	797	975	1.5736E-08	3.51957E-10
Ctnna1	catenin alpha-1	3518	4252	8521	8909	1.5701E-08	3.52455E-10
Mettl7a1	methyltransferase-like protein 7A	343	394	1053	706	1.6436E-08	3.56239E-10
H1f0	histone H1.0	3740	4305	8351	6570	1.8849E-08	4.32158E-10
Nrg4	pro-neuregulin-4, membrane-bound isoform	128	172	700	606	1.9428E-08	4.45623E-10
Zfp821	zinc finger protein 821	48	58	129	116	1.9453E-08	4.47008E-10
Ccl20	c-C motif chemokine 20	0	2	61	52	1.9595E-08	4.50615E-10
Dyrk2	dual specificity tyrosine-phosphorylation-regulated kinase	836	938	513	604	2.2246E-08	5.18777E-10
Krt7	keratin, type II cytoskeletal 7	36	22	429	362	2.3014E-08	5.39613E-10
Xkr8	XK-related protein 8	46	39	11	5	2.5402E-08	6.02318E-10
Ets1	protein C-ets-1	3567	4563	1262	1440	2.7036E-08	6.46358E-10
Ywhag	14-3-3 protein gamma	1930	2194	1161	1388	2.8235E-08	6.60561E-10
Sdf2	stromal cell-derived factor 2	528	669	1197	1086	2.9151E-08	6.8096E-10
Atp6ap1	ATPase, H%2B transporting, lysosomal accessory protein	2683	3266	7036	6565	3.0103E-08	6.8344E-10
Creb3l3	cAMP responsive element binding protein 3 3	3	3	27	25	3.0072E-08	7.26868E-10
C1rl	complement component 1, r subcomponent	11	11	73	43	3.1467E-08	7.6436E-10
Tlcd1	TLC domain-containing protein 1	208	227	684	660	3.4072E-08	8.2718E-10
Alyref	THO complex subunit 4	3601	4200	2119	2455	3.6069E-08	8.60473E-10
Lypd1	ly6/PLAUR domain-containing protein 1	72	73	165	148	3.6339E-08	8.95703E-10
Sertad2	SERTA domain-containing protein 2	2202	2708	3479	3892	3.7365E-08	9.23208E-10
Hist1h1c	histone H1.2	97	156	465	331	3.8549E-08	9.56073E-10
XPNPEP2	xaa-Pro aminopeptidase 2	3	1	24	22	4.4758E-08	1.1238E-09
Hnrnpa1	heterogeneous nuclear ribonucleoprotein A1	14120	16959	8343	9716	4.6277E-08	1.14415E-09
Actg2	actin, gamma-enteric smooth muscle, transcript variant 1	1404	1547	828	1030	4.9545E-08	1.25476E-09
Actg2	actin, gamma-enteric smooth muscle, transcript variant 2	1404	1547	828	1030	4.9545E-08	1.25497E-09
Chst14	carbohydrate sulfotransferase 14	833	970	570	599	4.9764E-08	1.26094E-09
Btg2	protein BTG2	11	11	119	114	5.1017E-08	1.29572E-09
Ppcs	phosphopantothenate--cysteine ligase	867	959	2021	2010	5.4165E-08	1.29628E-09
Vim	vimentin	46221	55291	21503	30032	5.7642E-08	1.3942E-09
Pdzrn3	PDZ domain containing RING finger 3	19	28	67	67	5.6314E-08	1.45232E-09
Pparg	Pparg	1310	1580	3339	2951	5.7967E-08	1.50025E-09
Prrg2	transmembrane gamma-carboxyglutamic acid protein 2	16	15	166	154	6.4592E-08	1.6415E-09
Cebpg	CCAAT/enhancer-binding protein gamma	183	240	405	359	6.5236E-08	1.71241E-09
Zeb1	zinc finger E-box-binding homeobox 1	1565	1959	332	339	6.5439E-08	1.71941E-09
PDP2	Pyruvate dehydrogenase [acetyl-transferring]-phosphatas	280	349	495	533	6.5455E-08	1.7204E-09
Galm	aldose 1-epimerase	435	458	1210	1183	6.8937E-08	1.82067E-09
Rps2	40S ribosomal protein S2	13087	14694	8184	10029	7.2564E-08	1.92516E-09
Exd1	exonuclease 3%27-5%27 domain containing 1	30	32	100	77	8.084E-08	2.16469E-09
Hook2	hook homolog 2	698	745	2825	2797	9.879E-08	2.45542E-09
Armcx5	armadillo repeat-containing X-linked protein 5	233	274	460	417	9.524E-08	2.59654E-09
Cnrip1	CB1 cannabinoid receptor-interacting protein 1	886	1067	504	486	9.8428E-08	2.59655E-09
Smad7	mothers against decapentaplegic homolog 7	21	41	2	5	1.0024E-07	2.74368E-09
Ppm1f	protein phosphatase 1F	1251	1324	451	518	1.0747E-07	2.95999E-09
Lama3	laminin, alpha 3	2	5	359	404	1.2491E-07	3.50264E-09
S100a10	protein S100-A10	2977	3571	1569	2205	1.3496E-07	3.62281E-09
Anxa1	annexin A1	7368	9092	13226	14992	1.4599E-07	3.82364E-09
Map6	microtubule-associated protein 6	2295	2261	1082	1513	1.6586E-07	4.77195E-09
SHISA4	protein shisa-4	14	18	115	112	1.8195E-07	4.951E-09
Taf7	Taf7	98	119	203	199	1.765E-07	5.10794E-09
Aplp2	amyloid-like protein 2	5971	7150	13412	12193	2.009E-07	5.45067E-09
Foxf2	forkhead box protein F2	415	421	791	738	1.9878E-07	5.82484E-09
Ppp1r3f	protein phosphatase 1 regulatory subunit 3F	131	150	252	247	2.0225E-07	5.94988E-09
NARS	asparaginyl-tRNA synthetase, cytoplasmic	1045	1287	1831	1756	2.1867E-07	6.49792E-09
Irf2bp1	interferon regulatory factor 2-binding protein	1354	1301	2084	2217	2.3342E-07	6.96425E-09
Cmah	cytidine monophospho-N-acetylneuraminic acid hydroxyla	272	317	173	172	2.3916E-07	7.16555E-09
Mmrn2	multimerin 2	1	4	21	24	2.4709E-07	7.43714E-09
Anxa4	annexin A4, transcript variant 1	1666	2098	4068	3928	2.7675E-07	7.58083E-09
Anxa4	annexin A4, transcript variant 2	1666	2098	4068	3928	2.7675E-07	7.58178E-09
Muc1	mucin-1	35	72	324	279	2.7204E-07	7.83933E-09
OLFM1	noelin, transcript variant 2	189	203	490	579	2.7648E-07	8.43718E-09
Abhd13	abhydrolase domain-containing protein 13	331	398	648	573	3.4305E-07	1.06906E-08
Fdft1	farnesyl diphosphate farnesyl transferase 1	1487	1544	2888	3223	4.7347E-07	1.51028E-08

Ddr1	discoidin domain receptor family, member 1, transcript va	1066	1047	3711	3742	4.7367E-07	1.52972E-08
Ddr1	discoidin domain receptor family, member 1, transcript va	1066	1047	3711	3743	4.739E-07	1.53086E-08
Rrs1	ribosome biogenesis regulatory protein homolog	1266	1259	515	659	4.8144E-07	1.55769E-08
Nfe2l1	nuclear factor, erythroid derived 2, 1, transcript variant 3	1673	1831	3512	3191	4.8368E-07	1.56607E-08
Nfe2l1	nuclear factor, erythroid derived 2, 1, transcript variant 4	1654	1814	3485	3149	4.8461E-07	1.56787E-08
Nfe2l1	nuclear factor, erythroid derived 2, 1, transcript variant 1	2341	2600	5094	4451	4.8527E-07	1.56868E-08
Nfe2l1	nuclear factor, erythroid derived 2, 1, transcript variant 2	2252	2493	4910	4276	4.8527E-07	1.56898E-08
Hsd3b2	3 beta-hydroxysteroid dehydrogenase/Delta 5-->4-isomer	12	18	58	46	4.8459E-07	1.57208E-08
Socs1	suppressor of cytokine signaling 1	288	297	151	188	5.4244E-07	1.77916E-08
Tmprss6	transmembrane serine protease 6	72	74	12	12	5.5054E-07	1.80899E-08
Ereg	proepiregulin	1477	1549	2599	3312	5.6994E-07	1.88003E-08
C1qbp	complement component 1 Q subcomponent-binding prote	2596	3212	1641	1844	6.2324E-07	1.98347E-08
Srd5a1	3-oxo-5-alpha-steroid 4-dehydrogenase 1	238	315	620	664	6.6866E-07	2.13172E-08
Amotl2	angiomotin 2	3090	3182	834	1013	6.3778E-07	2.13423E-08
Dnajb9	dnaJ homolog subfamily B member 9	218	284	553	529	6.5469E-07	2.19359E-08
Tagln2	transgelin-2	10281	11335	5182	6818	6.808E-07	2.28873E-08
Trim47	tripartite motif-containing protein 47	1820	2038	881	1127	7.1339E-07	2.31042E-08
Chst12	carbohydrate sulfotransferase 12	739	742	437	503	7.9775E-07	2.73951E-08
Tceal1	transcription elongation factor A protein 1	3	6	34	24	8.3835E-07	2.89395E-08
Cnn1	calponin-1	9	10	141	177	8.7278E-07	3.02527E-08
Sdcbp2	syntenin-2	19	11	233	181	9.8187E-07	3.32079E-08
Nufip2	nuclear fragile X mental retardation-interacting protein 2	5474	6668	3930	4347	1.0719E-06	3.50276E-08
Gpd1l	glycerol-3-phosphate dehydrogenase 1	835	787	1891	2041	1.0197E-06	3.58157E-08
Cisd2	CDGSH iron-sulfur domain-containing protein 1	300	392	667	658	1.0887E-06	3.85257E-08
Hspa8	Hspa8	64142	75883	31070	43140	1.0995E-06	3.89192E-08
Gpnmb	transmembrane glycoprotein NMB	1986	2326	525	581	1.1595E-06	4.12479E-08
Syt16	synaptotagmin XVI	86	85	361	359	1.2386E-06	4.43652E-08
Tssk2	testis-specific serine/threonine-protein kinase 3	10	15	38	46	1.492E-06	5.46935E-08
F8	coagulation factor VIII	12	16	47	43	1.6085E-06	5.95235E-08
Bcap31	B-cell receptor-associated protein 31	99	118	180	203	1.7504E-06	6.5343E-08
SHE	SH2 domain-containing adapter protein E	12	15	118	133	1.8534E-06	6.84649E-08
Hspe1	10 kDa heat shock protein, mitochondrial	6194	7524	4074	4910	2.1065E-06	7.83108E-08
PRR13	proline-rich protein 13	2440	2849	5560	5549	2.2088E-06	8.48984E-08
Chsy1	chondroitin sulfate synthase 1	1751	2101	1067	1333	2.2455E-06	8.59092E-08
Homez	homeobox and leucine zipper protein Homez	25	25	78	60	2.2358E-06	8.61486E-08
C3	complement C3	94	83	4325	2485	2.4141E-06	8.75933E-08
Nacad	NAC alpha domain containing	22	34	120	87	2.4841E-06	8.94151E-08
Bco2	beta-carotene oxygenase 2	150	186	966	788	2.7047E-06	1.03859E-07
Ypel5	protein yippee-like 5	210	272	579	501	2.7794E-06	1.09579E-07
OLFM1	noelin, transcript variant 1	781	872	2124	2587	3.1091E-06	1.24009E-07
Mex3d	RNA-binding protein MEX3D	791	730	386	453	3.4635E-06	1.39491E-07
Fam109a	sesquipedalian-1	134	151	82	67	3.4627E-06	1.40173E-07
Zfp365	protein ZNF365	22	39	3	8	3.5546E-06	1.44502E-07
FBL	*rRNA 2'-O-methyltransferase fibrillarin*	8852	9520	4341	6055	4.0727E-06	1.48639E-07
Tmbim4	transmembrane BAX inhibitor motif-containing protein 4	657	779	1478	1546	4.1513E-06	1.54399E-07
Fam46d	protein FAM46D	12	23	48	53	3.8926E-06	1.59733E-07
RRAGA	ras-related GTP-binding protein A	2101	2527	3075	3485	3.9102E-06	1.60488E-07
MAP3K3	mitogen-activated protein kinase 3	1045	1129	2317	2123	4.2001E-06	1.62722E-07
Srpx	sushi-repeat-containing protein	432	520	86	82	4.1818E-06	1.72755E-07
Pip4k2c	phosphatidylinositol-5-phosphate 4-kinase, type II, gamm	282	328	1041	1009	4.5361E-06	1.88781E-07
Dr1	protein Dr1	1583	2130	1107	1199	4.6376E-06	1.91783E-07
Slc5a2	solute carrier family 5 (sodium/glucose cotransporter), me	330	417	180	182	4.6022E-06	1.92809E-07
PLAC8	placenta-specific gene 8 protein	339	399	820	739	5.3691E-06	2.06925E-07
Naip1	NLR family, apoptosis inhibitory protein 1	10	10	37	34	5.0848E-06	2.15672E-07
Tcta	T-cell leukemia translocation-altered gene protein homolo	27	19	65	60	5.2071E-06	2.21429E-07
Tcta	T-cell leukemia translocation-altered gene protein homolo	27	19	65	60	5.2071E-06	2.21445E-07
Slc45a1	proton-associated sugar transporter A	448	525	1081	975	5.2954E-06	2.25671E-07
Zc3h6	zinc finger CCCH type containing 6	32	27	155	104	5.5111E-06	2.35766E-07
Cetn2	centrin-2	458	505	1116	1022	5.7028E-06	2.43272E-07
Lif	leukemia inhibitory factor	141	172	350	342	6.2745E-06	2.72598E-07
Rhou	rho-related GTP-binding protein RhoU	99	118	183	189	6.2943E-06	2.73511E-07
Taldo1	transaldolase	566	663	844	950	6.7244E-06	2.94778E-07
Zfp239	zinc finger protein 239	218	261	139	155	6.7527E-06	2.96535E-07
Sprr1b	cornifin-B	7	1	21	38	6.9171E-06	3.04111E-07
SELH	selenoprotein H	1610	1751	828	984	6.9414E-06	3.04127E-07
ZFR2	zinc finger RNA-binding protein 2	6	3	22	26	6.9308E-06	3.04828E-07
Abcd2	ATP-binding cassette, sub-family D %28ALD%29, membe	87	100	17	25	6.95E-06	3.05777E-07
Creld2	Creld2	2165	2304	3756	4367	7.4944E-06	3.07137E-07
PPP1R2	protein phosphatase inhibitor 2	1126	1516	2375	2535	7.8682E-06	3.14292E-07
Grina	glutamate [NMDA] receptor-associated protein 1	1253	1324	2450	2224	7.7222E-06	3.18225E-07

Cbx3	chromobox protein homolog 3	443	470	285	313	7.3025E-06	3.23417E-07
Tmem185b	transmembrane protein 185B	428	543	295	341	7.7032E-06	3.43261E-07
F2r	proteinase-activated receptor 1	2152	2544	1651	1738	7.9408E-06	3.55002E-07
Tmem140	transmembrane protein 140	49	56	125	99	8.023E-06	3.58948E-07
Zadh2	zinc-binding alcohol dehydrogenase domain-containing p	289	372	490	515	9.0349E-06	4.11837E-07
Tuba1c	Tuba1c	40378	45620	17145	24808	9.311E-06	4.25374E-07
Hspa1a	heat shock 70 kDa protein 1A	301	297	158	204	1.0373E-05	4.80962E-07
Sh3yl1	SH3 domain-containing YSC84-like protein 1	114	98	569	432	1.1861E-05	5.28006E-07
Fam110a	protein FAM110A	908	958	555	692	1.1299E-05	5.31006E-07
Bcap31	B-cell receptor-associated protein 31, transcript variant 1	1298	1542	2627	2641	1.185E-05	5.55569E-07
Zfp281	zinc finger protein 281	784	974	592	630	1.2793E-05	6.11856E-07
Cntnap2	contactin associated protein 2	9	4	46	50	1.3121E-05	6.29125E-07
Mars2	methionyl-tRNA synthetase, mitochondrial	1030	1105	734	755	1.3365E-05	6.43618E-07
Sc5d	lathosterol oxidase	3407	4123	6482	7273	1.4078E-05	6.8331E-07
CFH	complement factor H	25	45	370	265	1.4587E-05	6.94935E-07
Rras	ras-related protein R-Ras	491	560	1253	1288	1.4655E-05	7.14933E-07
ARF5	ADP-ribosylation factor 5	1456	1615	2440	2522	1.6289E-05	7.20646E-07
Tubal3	tubulin, alpha 3	5	4	24	22	1.5976E-05	7.86883E-07
Itfg3	protein ITFG3	2439	2709	5655	5200	1.6207E-05	7.93712E-07
Ckap4	cytoskeleton-associated protein 4	6092	6983	2887	4023	1.6416E-05	8.1092E-07
Tmbim6	bax inhibitor 1	6890	7989	12547	12351	1.7913E-05	8.4941E-07
Slc16a1	monocarboxylate transporter 1	4778	6036	3171	3850	1.9026E-05	8.80675E-07
PARP9	poly [ADP-ribose] polymerase 9	28	53	169	168	1.7678E-05	8.81382E-07
Map1lc3b	microtubule-associated proteins 1A/1B light chain 3B	2086	2639	5116	4584	1.9367E-05	9.04876E-07
Cisd3	CDGSH iron-sulfur domain-containing protein 3, mitochor	1511	1683	800	1032	1.8502E-05	9.28724E-07
Mre11a	meiotic recombination 11 homolog A	1714	2041	632	721	2.0224E-05	9.45204E-07
Harbi1	putative nuclease HARBI1	36	48	137	124	1.9403E-05	9.81945E-07
Orai3	protein orai-3	297	302	463	546	2.129E-05	1.02782E-06
Pcdhb3	protocadherin beta-3	324	353	497	516	2.1019E-05	1.07342E-06
Sema3e	sema domain, immunoglobulin domain, short basic doma	1397	1705	4715	4464	2.3042E-05	1.10686E-06
Zfp62	zinc finger protein 62	196	236	367	325	2.2701E-05	1.17319E-06
Tmem56	transmembrane protein 56	10	15	94	72	2.4649E-05	1.29277E-06
PLEKHF2	pleckstrin homology domain-containing family F member	214	237	427	390	2.4955E-05	1.29533E-06
Matn4	matrilin 4, transcript variant 2	2442	2729	1349	1537	2.5215E-05	1.30323E-06
Matn4	matrilin 4, transcript variant 1	2442	2729	1349	1537	2.5215E-05	1.30339E-06
Matn4	matrilin 4, transcript variant 3	2442	2729	1349	1537	2.5215E-05	1.30355E-06
Matn4	matrilin 4, transcript variant 4	2442	2729	1349	1537	2.5215E-05	1.30362E-06
HSPA1A	heat shock 70 kDa protein 1A/1B	294	295	144	208	2.4833E-05	1.30498E-06
S100a1	protein S100-A1	133	153	375	346	2.5273E-05	1.32301E-06
HCFC1R1	host cell factor C1 regulator 1, transcript variant 1	870	947	1561	1440	2.6523E-05	1.33021E-06
Rcc1	regulator of chromosome condensation	578	597	311	348	2.6383E-05	1.39983E-06
Dsel	dermatan sulfate epimerase	209	303	451	391	2.6649E-05	1.41495E-06
EXD1	Exonuclease 3'-5' domain-containing protein 1	16	18	56	42	2.8106E-05	1.49826E-06
ATOH8	protein atonal homolog 8	19	21	2	5	2.9689E-05	1.59683E-06
Gpr110	G protein-coupled receptor 110	33	34	272	316	3.5002E-05	1.67216E-06
Rpsa	40S ribosomal protein SA	12763	14332	9601	10430	3.3234E-05	1.73859E-06
Nucb2	nucleobindin-2	1733	2096	3351	3330	3.6514E-05	1.79383E-06
Atf5	cyclic AMP-dependent transcription factor ATF-5	2959	2856	5519	4960	3.4487E-05	1.9053E-06
Ldoc1l	protein LDOC1L	69	64	128	121	3.5555E-05	1.96958E-06
Srebf2	Srebf2	8809	9791	16075	16550	3.7186E-05	1.99101E-06
Tsta3	Tsta3	817	943	1867	2031	3.7024E-05	2.03199E-06
RRM2B	ribonucleoside-diphosphate reductase subunit M2	4708	5694	1908	3039	4.0381E-05	2.05244E-06
Apc2	adenomatosis polyposis coli 2	60	78	168	189	3.994E-05	2.25736E-06
Dpm3	Dpm3	127	140	77	77	4.0137E-05	2.27104E-06
Syne4	nesprin-4, transcript variant 1	33	36	282	201	4.2322E-05	2.28697E-06
Syne4	nesprin-4, transcript variant 2	32	33	248	167	4.2323E-05	2.2873E-06
Atxn7l3b	putative ataxin-7 protein 3B	1464	1808	2571	2269	4.0903E-05	2.32262E-06
Ppp1r3b	protein phosphatase 1 regulatory subunit 3B	60	72	136	113	4.6488E-05	2.69867E-06
Ccni	cyclin-I	2645	3271	5750	5829	4.7044E-05	2.74019E-06
Snx7	sorting nexin 7	496	640	1155	1282	4.7742E-05	2.75071E-06
Fth1	ferritin heavy chain	3167	4041	2495	2761	5.1758E-05	3.0482E-06
PPP1R14B	protein phosphatase 1 regulatory subunit 14B	5496	6374	3354	3940	5.4929E-05	3.25722E-06
B4galt5	B4galt5	379	519	982	979	6.5256E-05	3.3462E-06
PIGC	phosphatidylinositol N-acetylglucosaminyltransferase sub	203	245	391	327	6.0631E-05	3.638E-06
Tubg1	tubulin gamma-1 chain	2726	3032	1306	1724	6.7473E-05	3.87486E-06
Tomm5	mitochondrial import receptor subunit TOM5 homolog	1175	1381	871	814	6.775E-05	4.11235E-06
Arl1	XR_136082.1	793	986	1265	1240	6.8071E-05	4.14705E-06
Msln	mesothelin	75	77	723	556	7.0812E-05	4.33168E-06
Kifc2	kinesin family member C2	6	8	59	50	7.0891E-05	4.34663E-06
Nod2	nucleotide-binding oligomerization domain containing 2	23	22	59	50	7.5213E-05	4.64876E-06

Grcc10	protein C10	1044	1180	1874	1858	7.5262E-05	4.65129E-06
Slc39a1	zinc transporter ZIP1	1904	2094	3295	3448	8.2955E-05	4.80606E-06
PRCP	lysosomal Pro-X carboxypeptidase	726	869	1796	1692	8.7134E-05	4.82725E-06
Rasl10a	ras protein family member 10A	37	40	181	136	8.338E-05	4.94083E-06
Fzd7	frizzled-7	1106	1215	1660	1607	7.9597E-05	4.96247E-06
SBDS	ribosome maturation protein SBDS	566	714	1124	1127	8.4749E-05	5.03121E-06
TMEM66	transmembrane protein 66	1306	1615	2991	2740	8.4756E-05	5.05196E-06
Naga	alpha-N-acetylgalactosaminidase	1179	1286	2445	2310	9.2353E-05	5.08187E-06
Hist1h1b	histone H1.5	9	18	41	37	8.1664E-05	5.11637E-06
APBB2	amyloid beta A4 precursor protein-binding family B memb	31	41	70	76	8.6992E-05	5.50869E-06
Gnat1	guanine nucleotide binding protein, alpha transducing 1	148	146	400	390	9.3562E-05	5.54181E-06
Cyp51	lanosterol 14-alpha demethylase	7587	8777	15190	15354	9.2507E-05	5.54326E-06
Rabac1	PRA1 family protein 2	1091	1148	636	715	8.7608E-05	5.55157E-06
SSU72	RNA polymerase II subunit A C-terminal domain phospha	273	312	702	667	8.9643E-05	5.66014E-06
Vgf	neurosecretory protein VGF	656	620	196	310	8.9389E-05	5.69627E-06
Tuba1c	tubulin alpha-1C chain	49834	56713	21105	31833	8.9771E-05	5.72207E-06
Hprt1	hypoxanthine-guanine phosphoribosyltransferase	1202	1703	3058	3105	9.1814E-05	5.8256E-06
Naip2	NLR family, apoptosis inhibitory protein 2	6	8	26	25	0.00010023	6.51414E-06
Lrrc59	leucine-rich repeat-containing protein 59	7246	8443	4171	5568	0.00012173	6.96467E-06
Hmga1	High mobility group protein HMG-I/HMG-Y	1473	1376	666	953	0.00010734	6.99091E-06
Zfp184	Zinc Finger Protein 184 (Kruppel-Like)	157	220	609	470	0.00010916	7.16832E-06
Nfkbiz	nuclear factor of kappa light polypeptide gene enhancer ir	13	10	74	56	0.00010988	7.23304E-06
Slc25a20	mitochondrial carnitine/acylcarnitine carrier protein	1327	1480	3110	2636	0.00011177	7.2772E-06
Lmo2	rhombotin-2	160	200	543	463	0.00011183	7.37678E-06
Exph5	exophilin 5	160	219	1038	1031	0.0001221	8.15275E-06
HDHD3	haloacid dehalogenase hydrolase domain-containing prot	16	24	43	55	0.00012327	8.24502E-06
Syde1	synapse defective 1, Rho GTPase, homolog 1 %28C. ele	889	921	309	337	0.00012672	8.45047E-06
Aga	n(4)-(beta-n-acetylglucosaminyl)-l-asparaginase, transcrip	2495	2777	4649	4408	0.00012724	8.47244E-06
Arsa	arylsulfatase A	652	669	1811	1602	0.00012909	8.6716E-06
Zbtb5	zinc finger and BTB domain-containing protein 5	97	103	177	159	0.0001294	8.72453E-06
Sepw1	selenoprotein W	480	613	273	335	0.00013723	8.86947E-06
ITGA3	integrin alpha-3	1019	1065	3337	3692	0.00013501	8.98905E-06
LIG4	DNA ligase 4	303	407	223	250	0.00013294	9.01065E-06
Cyb5r3	NADH-cytochrome b5 reductase 3	6009	6778	10899	10392	0.00014435	9.09954E-06
Tigd2	tigger transposable element-derived protein 2	278	337	450	445	0.00013501	9.16567E-06
Fra-2	fos-related antigen 2	313	341	885	737	0.00013768	9.38089E-06
Rps2	Rps2	61611	64802	36225	45099	0.00014688	9.50832E-06
Notum	notum pectinacetylesterase homolog %28Drosophila%29	6	8	34	22	0.0001403	9.59188E-06
Tpbg	trophoblast glycoprotein	918	1077	1271	1469	0.00014296	9.7953E-06
Idi1	isopentenyl-diphosphate Delta-isomerase 1	2214	2816	6966	7995	0.00014442	9.91022E-06
Clstn1	calsyntenin 1	2739	3024	5520	5074	0.000172	1.02292E-05
Ttll11	tubulin polyglutamylase TTLL11	2	4	23	14	0.00016343	1.14641E-05
U2af2	splicing factor U2AF 65 kDa subunit	1127	1298	807	964	0.00016554	1.16333E-05
UBE2C	ubiquitin-conjugating enzyme E2 C	3472	3840	1798	2524	0.00019065	1.1759E-05
HIST1H2BN	histone H2B type 1-N	257	351	470	438	0.00016922	1.19346E-05
Lcp1	lymphocyte cytosolic protein 1	12	13	126	96	0.00019503	1.26073E-05
Zhx2	zinc fingers and homeoboxes protein 2	1404	1540	1941	2041	0.00018093	1.29429E-05
INSIG2	insulin-induced gene 2 protein	52	53	89	102	0.00018569	1.33416E-05
HNRNPM	heterogeneous nuclear ribonucleoprotein M	6692	7168	4033	5244	0.0002166	1.33549E-05
HBP1	HMG box-containing protein 1	1216	1563	3058	2776	0.00018797	1.33986E-05
TRAF7	E3 ubiquitin-protein ligase TRAF7	497	566	709	766	0.00018779	1.351E-05
EPHX1	epoxide hydrolase 1	5557	6254	12140	10660	0.00018878	1.36081E-05
Eif2c2	eukaryotic translation initiation factor 2C, 2	2577	3057	965	1271	0.00020237	1.36435E-05
ZBTB26	zinc finger and BTB domain-containing protein 26, transcr	101	128	184	180	0.00019828	1.43799E-05
Dbil5	Diazepam-binding inhibitor-like 5	67	80	39	37	0.00019868	1.44155E-05
ATP6V1F	V-type proton ATPase subunit F	980	1137	1549	1488	0.00021279	1.45228E-05
Fam89b	protein FAM89B, transcript variant 1	1822	1833	1239	1327	0.0002159	1.47945E-05
AGA	N(4)-(beta-N-acetylglucosaminyl)-L-asparaginase, transcr	2813	3281	5387	5090	0.00020489	1.48861E-05
Ccdc85b	coiled-coil domain-containing protein 85B	4733	4280	2613	3369	0.00020646	1.50537E-05
B4galt3	B4galt3	427	469	961	891	0.00024126	1.53335E-05
Dgat2	diacylglycerol O-acyltransferase 2	194	199	676	612	0.00023722	1.59418E-05
DLC1	rho GTPase-activating protein 7, transcript variant 2	246	326	32	54	0.00024465	1.61725E-05
DLC1	rho GTPase-activating protein 7, transcript variant 1	317	407	46	73	0.00024469	1.61764E-05
Olfr1002	olfactory receptor 1002	108	116	228	169	0.00022236	1.64022E-05
Nit1	nitrilase homolog 1	1005	1206	2166	1947	0.00025257	1.69806E-05
Cyp4f14	leukotriene-B4 omega-hydroxylase 3	8	2	20	23	0.00023722	1.7695E-05
Tmem176b	transmembrane protein 176B	275	308	880	830	0.00024252	1.77622E-05
Slc25a1	tricarboxylate transport protein, mitochondrial	977	956	2193	2282	0.0002386	1.78101E-05
Stc2	stanniocalcin-2	701	778	1420	1311	0.00025655	1.84001E-05
Sqstm1	sequestosome 1	3270	3778	6726	5570	0.00028718	1.85635E-05

UBE2S	ubiquitin-conjugating enzyme E2 S	7755	7996	4324	5937	0.00026928	1.91197E-05
Hist1h1d	histone H1.3	13	14	41	33	0.00026084	1.97234E-05
Crebzf	CREB/ATF bZIP transcription factor	488	578	815	725	0.00026232	1.98577E-05
Tmem165	transmembrane protein 165	557	747	1095	1146	0.00029317	2.03828E-05
Lrp10	low-density lipoprotein receptor-related protein 10	2837	3084	6692	6560	0.00030192	2.09937E-05
Mtbp	mdm2-binding protein	1016	1099	622	722	0.00028407	2.14982E-05
Rpsa	Rpsa	43667	48300	32244	35354	0.00034791	2.15713E-05
SH3BGRL	SH3 domain-binding glutamic acid-rich-like protein	4245	5563	8859	8050	0.00031182	2.21127E-05
SH3BGRL3	SH3 domain-binding glutamic acid-rich-likr protein 3	701	747	354	503	0.0003197	2.3065E-05
Pink1	PTEN induced putative kinase 1	536	495	1076	1065	0.00034217	2.37575E-05
Stx11	syntaxin-11	346	410	250	281	0.00030782	2.39967E-05
Abtb2	ankyrin repeat and BTB %28POZ%29 domain containing	34	32	191	184	0.00032973	2.43931E-05
Vamp5	vesicle-associated membrane protein 5	42	35	123	119	0.00032563	2.56158E-05
Zfp583	zinc finger protein 583	9	9	38	24	0.00035214	2.80995E-05
Spry4	protein sprouty homolog 4	23	30	7	10	0.00036011	2.88734E-05
PLD3	phospholipase D3	654	688	1650	1568	0.0004249	2.96557E-05
Tspan6	tetraspanin-6	1479	1925	2970	2747	0.00044984	3.10226E-05
DHCR24	delta(24)-sterol reductase	5764	6378	9217	11093	0.00040667	3.13114E-05
PSAT1	phosphoserine aminotransferase	919	1052	1488	1434	0.00039042	3.17083E-05
Tmem176a	transmembrane protein 176A	195	187	658	563	0.00039591	3.18797E-05
ELOVL7	elongation of very long chain fatty acids protein 7	349	440	895	899	0.00043687	3.22802E-05
Rbm14	RNA binding motif protein 14	2147	2257	1128	1596	0.00044007	3.38796E-05
Npas1	neuronal PAS domain-containing protein 1	14	13	33	36	0.00042618	3.53687E-05
Gata2	GATA binding protein 2, transcript variant 2	490	556	230	267	0.00045609	3.56693E-05
Ssr4	translocon-associated protein subunit delta	1626	1969	2733	2654	0.00052176	3.63256E-05
Glce	glucuronyl C5-epimerase, transcript variant 1	466	630	920	989	0.00045402	3.81107E-05
Glce	glucuronyl C5-epimerase, transcript variant 2	466	630	920	989	0.00045419	3.8132E-05
Ptgfrn	prostaglandin F2 receptor negative regulator	7777	9302	12528	13011	0.00053395	3.81827E-05
FAM89B	protein FAM89B, transcript variant 2	945	896	571	631	0.00047432	3.84159E-05
Letm2	leucine zipper-EF-hand containing transmembrane protei	11	7	28	27	0.00046135	3.88493E-05
Asah1	acid ceramidase	1333	1607	2757	2623	0.00058623	4.01647E-05
Ngef	ephexin-1	26	21	6	7	0.00048834	4.15252E-05
Plin4	perilipin-4	750	663	3618	2826	0.00051986	4.20404E-05
Mpv17	mpv17 protein	40	34	121	117	0.00051924	4.33225E-05
Cr1l	complement regulatory protein Crry	1988	2365	3944	4232	0.00050791	4.33258E-05
Agrn	agrin, transcript variant 1	3292	3656	8468	8995	0.00052508	4.4421E-05
Tspan3	tetraspanin-3	4517	5551	7615	7759	0.00055938	4.61761E-05
Eid1	EP300-interacting inhibitor of differentiation 1	1365	1534	1103	1053	0.00056599	4.9391E-05
Sacs	sacsin	2133	2783	1201	1439	0.00057224	5.00778E-05
STEAP1	metalloreductase STEAP1	585	694	1130	1074	0.00060153	5.03743E-05
Zbtb7b	zinc finger and BTB domain-containing protein 7B	266	302	546	514	0.00059791	5.26791E-05
Slc6a9	sodium- and chloride-dependent glycine transporter 1	403	417	1276	997	0.0006292	5.29272E-05
Plk2	serine/threonine-protein kinase PLK2	1097	1335	3154	2532	0.00062691	5.42339E-05
Sept1	septin-1	26	23	113	88	0.00066749	5.48339E-05
Glis2	GLIS family zinc finger 2	868	990	433	497	0.00064818	5.55334E-05
Sf3b5	splicing factor 3B subunit 5	2625	2552	1849	1949	0.00065187	5.81407E-05
ARL2BP	ADP-ribosylation factor protein 2-binding protein	2835	3680	1288	1540	0.00074319	5.83278E-05
GYPC	glycophorin-C	152	177	104	112	0.00065384	5.83329E-05
TRAF1	TNF receptor-associated factor 1	260	328	67	101	0.00067416	6.00058E-05
ABCF2	ATP-binding cassette sub-family F member 2	1241	1411	923	1083	0.00069273	6.25048E-05
Zbed4	zinc finger, BED domain containing 4	458	550	348	389	0.00070265	6.3549E-05
Zfp786	zinc finger protein 786	16	18	47	36	0.00071812	6.51368E-05
Hist1h2bc	histone h2b type 1-c/e/g, transcript variant 2	337	446	567	538	0.00071876	6.52327E-05
Tubb3	tubulin beta-3 chain	90	93	49	58	0.00072834	6.62141E-05
Rptn	repetin	15	4	49	28	0.00073008	6.64215E-05
Pxmp3	peroxisomal membrane protein 3	55	80	102	125	0.00073947	6.74148E-05
CCDC124	coiled-coil domain-containing protein 124	2076	2194	1295	1586	0.00082877	6.74488E-05
Rcbtb1	regulator of chromosome condensation and BTB (POZ) d	596	750	1464	1353	0.00089365	6.74606E-05
Abhd4	abhydrolase domain containing 4	955	1211	2750	2670	0.00076481	6.88955E-05
Tmed4	transmembrane emp24 domain-containing protein 4	1365	1617	2508	2519	0.00084391	6.90719E-05
Acp5	acid phosphatase 5, tartrate resistant	96	92	327	254	0.00076473	7.01707E-05
Ppp1r15a	protein phosphatase 1 regulatory subunit 15A	884	1028	2031	1430	0.00077475	7.09119E-05
GLTPD1	glycolipid transfer protein domain-containing protein 1	228	249	479	446	0.0008699	7.36847E-05
Tex14	testis-expressed protein 14	3	4	22	13	0.00080466	7.46801E-05
Gjb5	gap junction beta-5 protein	1481	1852	1003	1268	0.00082208	7.51512E-05
Lmna	prelamin-A/C, transcript variant 3	11136	11963	6695	8409	0.000898	7.54789E-05
Ctsl	cathepsin L1	5335	6788	10724	10573	0.00081566	7.58926E-05
Il4i1	interleukin 4 induced 1	42	40	88	71	0.00083424	7.79938E-05
Rprm	protein reprimo	205	246	294	357	0.0008419	7.88819E-05
LTBP1	latent-transforming growth factor beta-binding protein 1	1045	1198	132	243	0.00096544	7.92429E-05

Lat2	linker for activation of T-cells family member 2	6	8	21	24	0.00086234	8.10696E-05
Lmna	prelamin-A/C, transcript variant 2	11422	12270	6875	8621	0.00099499	8.17298E-05
Lmna	prelamin-A/C, transcript variant 1	11421	12269	6875	8620	0.00099508	8.17411E-05
Csnk1g2	casein kinase I isoform gamma-2	2301	2248	3815	3965	0.001065	8.25616E-05
RGS16	regulator of G-protein signaling 16	46	31	115	116	0.00100004	8.37199E-05
Ntn4	netrin 4	686	842	2192	2197	0.00093832	8.93703E-05
HIST1H2BK	histone H2B type 1-K	683	886	1141	1085	0.00104077	9.04023E-05
HMOX1	heme oxygenase 1	2350	2932	1505	1935	0.00110301	9.22914E-05
Cdh9	cadherin 9	16	8	93	60	0.00097735	9.29591E-05
Man2b2	mannosidase 2, alpha B2, transcript variant 1	1187	1347	3079	2600	0.00117358	9.31545E-05
Man2b2	mannosidase 2, alpha B2, transcript variant 2	1102	1262	2838	2380	0.00118755	9.38436E-05
HOXA9	homeobox protein Hox-A9	33	52	104	70	0.00098103	9.42078E-05
Man2b2	mannosidase 2, alpha B2, transcript variant 3	1094	1256	2836	2370	0.00118976	9.43155E-05
HECA	headcase protein homolog	426	436	833	775	0.00102434	9.91322E-05
Nmnat2	nicotinamide nucleotide adenylyltransferase 2	3	10	50	63	0.0010912	0.000106878
Ulk3	serine/threonine-protein kinase ULK3	54	41	129	100	0.00114527	0.000107767
Sesn2	sestrin 2	215	208	1079	786	0.00122483	0.000108308
Serpinf1	pigment epithelium-derived factor	66	57	419	194	0.00111541	0.00010857
Timm13	mitochondrial import inner membrane translocase subunit	4061	4045	2610	2882	0.00123797	0.000108796
Siae	sialate O-acetylesterase	513	627	1363	1155	0.00130269	0.000109005
Cyb5r3	NADH-cytochrome b5 reductase	6	7	23	20	0.0011262	0.000111093
Neu1	Neu1	260	253	638	563	0.00115181	0.0001124
Tgif1	homeobox protein TGIF1, transcript variant 2	581	707	1049	985	0.00117229	0.00011471
AVPI1	arginine vasopressin-induced protein 1	583	564	1118	1189	0.0011594	0.000115058
Zc2hc1c	protein FAM164C	32	41	70	65	0.00116952	0.000116223
Lsm3	U6 snRNA-associated Sm protein LSm3	1289	1569	785	1004	0.0013999	0.000119976
Ssh3	protein phosphatase Slingshot homolog 3	213	206	856	876	0.00142401	0.00012016
Nucb1	nucleobindin-1	1391	1530	2597	2773	0.0012526	0.000121493
CD59	CD59 glycoprotein	546	703	1707	1659	0.00123131	0.000123513
CDKN2C	cyclin-dependent kinase 4 inhibitor C	445	504	860	675	0.00123349	0.000123591
Hspa5	Hspa5	26369	29756	45602	54440	0.00134928	0.000124178
Irs2	insulin receptor substrate 2	249	236	388	431	0.00124007	0.000124368
Pcna	Pcna	6596	8794	3697	5165	0.00125413	0.000124602
Ncs1	neuronal calcium sensor 1	276	270	89	96	0.00136629	0.000125637
wrb	tryptophan-rich protein	887	1008	449	493	0.00126903	0.000127857
Ngf	beta-nerve growth factor	38	44	18	20	0.00127137	0.000128222
JRK	jerky protein homolog	172	210	263	282	0.00127994	0.00012932
Gata2	GATA binding protein 2, transcript variant 1	509	580	250	289	0.0014247	0.000132026
Mybl2	myeloblastosis oncogene 2	8632	9297	4548	6425	0.00155843	0.000136209
Wdtc1	WD and tetratricopeptide repeats 1	1005	1146	2186	2174	0.00155727	0.000142834
Lrrc8b	leucine rich repeat containing 8 family, member B	396	468	897	905	0.00142667	0.000145476
CRIP2	hydrogen peroxide-induced mRNA	3233	4272	7877	5382	0.00151863	0.000145488
Txnip	thioredoxin-interacting protein	1457	1644	2969	2450	0.00170978	0.000146538
FUS	RNA-binding protein FUS	12413	13254	7068	8707	0.00166968	0.000147489
PCDHB5	protocadherin beta-5	68	92	134	123	0.00145206	0.000150336
Lpar6	lysophosphatidic acid receptor 6	139	183	257	224	0.00147021	0.000152541
Arhgap32	rho GTPase-activating protein 8	34	33	133	118	0.001587	0.000157923
sdh	probable saccharopine dehydrogenase	586	730	1441	1262	0.00174836	0.000158274
Acsl4	long-chain-fatty-acid--CoA ligase 4	1835	2353	3775	3755	0.00180792	0.000164443
HERC6	probable E3 ubiquitin-protein ligase HERC6	135	141	91	89	0.0016391	0.00017347
Fam187a	Ig V-type domain-containing protein FAM187A	40	29	81	62	0.00164345	0.000174071
MARCH9	E3 ubiquitin-protein ligase MARCH9	748	626	851	912	0.00173499	0.000175949
Dbp	D site-binding protein	79	93	211	170	0.00170058	0.000181526
Etl4	enhancer trap locus 4	4	11	21	25	0.00174293	0.000186908
Gjb3	gap junction beta-3 protein	1855	2158	1037	1609	0.00194531	0.000187191
Fzd4	frizzled-4	192	224	90	105	0.00180533	0.000195122
Mesdc2	LDLR chaperone MESD	2875	3410	4498	4655	0.00191699	0.000209491
Nppb	natriuretic peptides B	208	267	65	141	0.00215349	0.000211215
Fam101b	protein FAM101B	987	1056	460	587	0.00194108	0.000212931
Pgrmc1	membrane-associated progesterone receptor component	1366	1612	2303	2231	0.00197481	0.000217464
Nxt1	NTF2-related export protein 1, transcript variant 2	448	513	342	378	0.00198522	0.000219281
Lepre1	prolyl 3-hydroxylase 1	990	1117	2743	2471	0.00200732	0.000220129
Bbs12	Bardet-Biedl syndrome 12 protein homolog	114	156	188	208	0.00201328	0.000223136
Litaf	lipopolysaccharide-induced tumor necrosis factor-alpha fa	2108	2431	3457	3283	0.00222031	0.000233036
Zfp90	zinc finger protein 90	1	8	16	19	0.00215638	0.000243073
Dnase1l1	Dnase1l1	58	74	212	191	0.00239726	0.000249472
Tardbp	TAR DNA-binding protein 43, transcript variant 2	2739	3255	1989	2165	0.00261979	0.000250178
Kpnb1	karyopherin %28importin%29 beta 1	19329	22843	12091	14597	0.00227177	0.000254855
Fam198b	protein FAM198B	464	686	267	277	0.00241035	0.000259557
Nxt1	NTF2-related export protein 1, transcript variant 1	498	555	363	394	0.00252563	0.000261043

Tardbp	TAR DNA-binding protein 43, transcript variant 3	2552	3058	1856	2011	0.00275646	0.000261535
Pcdhb7	protocadherin beta-7	116	129	173	187	0.0023261	0.000267295
Plch2	phospholipase C, eta 2	8	7	28	19	0.00233064	0.000267937
S1pr2	sphingosine-1-phosphate receptor 2	2129	2428	1756	1863	0.00237866	0.000274227
ACAT2	acetyl-CoA acetyltransferase, cytosolic	8300	9297	19959	22112	0.00241408	0.000278981
Efhd2	EF-hand domain-containing protein D2	3764	4371	2082	2799	0.00273699	0.000286674
Hmgb1	high mobility group protein B1	10461	12400	7169	8864	0.00292183	0.000293188
Tmem123	porimin	424	565	876	832	0.00256186	0.000300266
Ccne1	G1/S-specific cyclin-E1	683	923	386	414	0.00297414	0.000301156
Tardbp	TAR DNA-binding protein 43, transcript variant 1	4912	5875	3543	3922	0.00316527	0.000301329
Jrk	jerky protein	150	170	217	239	0.00256989	0.000301482
Egr2	early growth response protein 2	49	44	77	84	0.00264455	0.000312381
Spry1	protein sprouty homolog 1	31	10	55	55	0.00268204	0.000317659
Pycr1	pyrroline-5-carboxylate reductase 1, mitochondrial	264	256	618	548	0.00313009	0.000329608
Hmgn1	non-histone chromosomal protein HMG-14	2260	2503	1363	1699	0.00279242	0.000332178
Atp6v0d1	ATPase, H%2B transporting, lysosomal V0 subunit D1	1465	1565	2722	2540	0.00297173	0.000333479
Myom3	myomesin family, member 3	1	9	26	15	0.00279301	0.000333747
Pcdhb10	protocadherin beta-10	131	166	202	223	0.00279612	0.000334286
Stbd1	starch-binding domain-containing protein 1	77	76	161	109	0.00282248	0.00033811
Eif6	eukaryotic translation initiation factor 6	7512	8561	4623	5630	0.00302739	0.000348622
Zmat4	zinc finger matrin-type protein 4	14	12	49	51	0.00291633	0.000352083
Chmp2b	charged multivesicular body protein 2b	767	1135	1637	1711	0.00353306	0.000355321
P2ry2	purinergic receptor P2Y, G-protein coupled 2	268	326	222	204	0.00296607	0.000359126
Atf3	cyclic AMP-dependent transcription factor ATF-3	27	41	108	105	0.00301506	0.000362156
Wdr89	WD repeat-containing protein 89	196	225	130	165	0.0030037	0.000364654
Fth1	ferritin heavy chain 1	27027	31880	19571	21809	0.00303567	0.000366637
Ugp2	Ugp2	748	977	1910	1724	0.00322029	0.000367851
Cd151	CD151 antigen	3701	4276	6055	6570	0.00334699	0.000368867
Chac1	cation transport regulator protein 1	30	38	104	76	0.00306472	0.000369539
AOC1	amiloride-sensitive amine oxidase [copper-containing]	297	339	82	88	0.00311429	0.000369647
Mpzl3	myelin protein zero-like protein 3	4	5	14	19	0.00307822	0.000375417
Bambi	BMP and activin membrane-bound inhibitor homolog	234	263	152	171	0.00350799	0.00037891
Psph	phosphoserine phosphatase	1230	1469	2163	2121	0.00363501	0.000383428
Ncl	nucleolin	34877	40275	24054	28132	0.00401578	0.000385404
Klhl9	kelch protein 9	1422	1810	2098	2077	0.00318778	0.000391834
MOAP1	modulator of apoptosis 1	422	489	678	582	0.00332093	0.000411567
Atpif1	ATPase inhibitor, mitochondrial	1069	1125	1717	1976	0.00339322	0.000413749
Aftph	aftiphilin	755	910	1971	1752	0.00372576	0.000417077
Ghitm	growth hormone-inducible transmembrane protein	6998	8574	14047	13325	0.00340564	0.00042447
Elavl2	ELAV like neuron-specific RNA binding protein 2, transcri	121	179	36	45	0.00386763	0.000427911
Setmar	histone-lysine N-methyltransferase SETMAR	58	58	120	86	0.00343738	0.000429394
Elavl2	ELAV like neuron-specific RNA binding protein 2, transcri	142	200	41	48	0.00390403	0.000430077
Spsb2	SPRY domain-containing SOCS box protein 2	181	191	276	299	0.00396477	0.000439111
Carns1	carnosine synthase 1	133	142	350	354	0.00352722	0.00044246
Cdc6	cell division cycle 6 homolog %28S. cerevisiae%29	983	1124	402	564	0.00424166	0.000443635
Spry2	protein sprouty homolog 2	239	270	372	336	0.00356529	0.000449016
C1qtnf2	complement C1q tumor necrosis factor-related protein 2	37	30	13	16	0.00360228	0.000454566
Bscl2	seipin	59	82	372	289	0.00435118	0.000483863
Bola1	bolA protein 1	445	435	324	323	0.00383378	0.000490178
Enox2	ecto-NOX disulfide-thiol exchanger 2	7	6	18	21	0.00391219	0.000502002
Ccrn4l	nocturnin	1078	1262	1777	1807	0.00396848	0.000510759
Txnl4a	thioredoxin protein 4A	628	764	508	569	0.00398213	0.000512978
Ranbp1	ran-specific GTPase-activating protein	4329	5120	2689	3363	0.00403813	0.000521323
Tmem126b	transmembrane protein 126B	699	861	1260	1213	0.00472092	0.000524539
Zeb1	zinc finger E-box-binding homeobox 2	1097	1391	421	390	0.00407377	0.000525423
Smyd1	SET and MYND domain containing 1	13	21	74	96	0.00468402	0.000528919
NPC2	epididymal secretory protein E1	5041	5994	8543	8320	0.00433196	0.000548202
Smpd1	sphingomyelin phosphodiesterase 1, acid lysosomal	2150	2256	3175	3241	0.00504119	0.000550531
Ifitm2	interferon-induced transmembrane protein 2	12337	14215	17398	17376	0.00487605	0.000551305
Zmiz2	zinc finger, MIZ-type containing 2, transcript variant 1	750	781	1789	1800	0.00538312	0.000569226
Gbgt1	globoside alpha-1,3-N-acetylgalactosaminyltransferase 1	50	83	157	146	0.00439199	0.000577603
Tlr1	toll-like receptor 1	38	32	80	57	0.00443696	0.000585046
Pnpla2	patatin phospholipase domain containing 2, transcript var	497	529	1415	1339	0.00460366	0.000593075
Pnpla2	patatin phospholipase domain containing 2, transcript var	572	608	1627	1553	0.00464289	0.000596102
Zmiz2	zinc finger, MIZ-type containing 2, transcript variant 2	740	766	1766	1771	0.00571433	0.000596467
Elavl2	ELAV like neuron-specific RNA binding protein 2, transcri	47	79	16	19	0.00551867	0.00060304
Sfxn1	sideroflexin 1	6884	8285	3800	4313	0.00483112	0.000617672
Phf21b	PHD finger protein 21B	343	373	82	88	0.0047024	0.000624196
Gpr146	probable G-protein coupled receptor 146	18	15	34	36	0.00470293	0.000628857
St3gal1	CMP-N-acetylneuraminate-beta-galactosamide-alpha-2,3	336	413	174	183	0.00545776	0.000680074

Eif4a1	eukaryotic initiation factor 4A-I	3916	4753	3271	3711	0.00509992	0.000693066
Mob3b	mps one binder kinase activator-like 2B	116	146	220	203	0.00540231	0.000693212
Ndfip1	NEDD4 family-interacting protein 1	1212	1501	2361	2259	0.0057728	0.000694325
Pcdhb18	protocadherin beta-18	116	141	171	195	0.00512223	0.000696753
Akap2	A kinase %28PRKA%29 anchor protein 2	1036	1144	448	473	0.00562945	0.000700668
Zfp397	zinc finger protein 397	31	31	76	66	0.00577528	0.000707179
Bnip3	BCL2/adenovirus E1B 19 kDa protein-interacting protein 3	1545	2012	2850	3250	0.00599641	0.000713566
Thbs3	thrombospondin 3	919	966	2258	2023	0.00700224	0.000717498
Tmem63a	transmembrane protein 63a	177	179	931	822	0.00626082	0.00072228
Zfp260	zinc finger protein 260	353	462	624	514	0.00548418	0.000756277
Per3	period circadian protein homolog 3	72	72	139	124	0.00591185	0.00077814
Tbc1d10c	TBC1 domain family, member 10c	50	39	185	200	0.00668949	0.000796199
Ninj2	ninjurin-2	32	24	84	76	0.00653478	0.000813623
Kpna2	karyopherin %28importin%29 alpha 2	6821	8452	4264	5777	0.00702429	0.000823428
Serinc3	serine incorporator 3	1608	1921	3336	3051	0.00616781	0.000844609
Cebpd	CCAAT/enhancer-binding protein delta	192	165	250	268	0.00601371	0.000847057
Tnfaip8l1	tumor necrosis factor alpha-induced protein 8-like protein	44	46	22	26	0.0062704	0.000891598
Birc5	baculoviral IAP repeat-containing protein 5	402	479	253	319	0.00630545	0.000895119
Rwdd3	RWD domain-containing protein 3	18	33	70	58	0.00704395	0.000899893
Ssbp2	single-stranded DNA binding protein 2	30	30	57	51	0.00635069	0.000906423
Tpt1	translationally-controlled tumor protein	3777	4502	5631	5458	0.00656186	0.000911745
Lpl	Lpl	4728	5926	9521	8063	0.00795686	0.000916867
Samd12	sterile alpha motif domain-containing protein 12	56	50	228	173	0.00679603	0.000920453
Klhl38	kelch 38 %28Drosophila%29	36	27	63	55	0.00647309	0.000929074
Arf3	ADP-ribosylation factor 3	1787	2038	2864	2788	0.00672617	0.00093926
Rcc1	regulator of chromosome condensation, transcript variant	3355	3749	1729	2154	0.00682236	0.000944697
Rcc1	regulator of chromosome condensation, transcript variant	3355	3749	1729	2154	0.00682287	0.000944715
Rcc1	regulator of chromosome condensation, transcript variant	3355	3749	1729	2154	0.00682277	0.000944797
Ccdc71	coiled-coil domain-containing protein 71	546	570	842	702	0.00660141	0.00095199
Sat1	spermidine/spermine N1-acetyltransferase	1030	1337	2115	2000	0.00746864	0.000957674
FCGBP	IgGFc-binding protein	19	19	4	9	0.00673489	0.000976287
Tmem30a	transmembrane protein 30A	1244	1710	2703	2702	0.00801923	0.000990482
Zcchc24	zinc finger CCHC domain-containing protein 24	547	659	212	273	0.00756081	0.000998351
Dnajb4	DnaJ %28Hsp40%29 homolog, subfamily B, member 4	242	324	582	487	0.0069016	0.001005761
Ctla2b	protein CTLA-2-beta	2885	3851	6557	6171	0.00693159	0.001012199
Carhsp1	calcium-regulated heat stable protein 1	972	986	1752	1962	0.00720835	0.001031937
Slc45a1	solute carrier family 45, member 1	613	579	1196	1134	0.0070447	0.001032717
Nudt12	nudix %28nucleoside diphosphate linked moiety X%29-ty	127	150	428	289	0.00744672	0.001037568
Lpcat3	lysophospholipid acyltransferase 5	2362	2506	3875	3833	0.00780066	0.001043163
Cmtm4	CKLF-like MARVEL transmembrane domain-containing p	97	126	197	221	0.00749399	0.001050263
Gpr6	G-protein coupled receptor 6	17	20	6	7	0.00717083	0.001055665
Fcer2a	low affinity immunoglobulin epsilon Fc receptor, transcript	23	22	41	44	0.00719191	0.001059688
Pdlim2	PDZ and LIM domain protein 2, transcript variant 1	17	11	53	38	0.00755469	0.00106991
H2afz	histone H2A.Z	3215	3708	2046	2865	0.00818859	0.001084165
Neu2	Neu2	881	1032	544	639	0.00737445	0.001091859
Ppp1r18	phostensin	1547	1558	662	853	0.00744793	0.001105632
Ppp1cb	serine/threonine-protein phosphatase PP1-beta catalytic s	3452	4726	5909	5757	0.00982116	0.001123172
Igfbp4	insulin growth factor-binding protein 4	4419	4100	2404	3121	0.00786211	0.001126872
KTI12	protein KTI12 homolog	1219	1269	949	1023	0.00759457	0.0011337
Myl12b	myosin regulatory light chain 12B	11199	12975	15795	16442	0.00771435	0.001147843
S100a3	protein S100-A3	79	83	32	47	0.00861951	0.00116898
Evpl	envoplakin	31	36	63	54	0.00781799	0.001174753
Hoxa4	homeobox protein Hox-A4	64	76	126	97	0.00788582	0.001187425
Plcg1	1-phosphatidylinositol-4,5-bisphosphate phosphodiestera	5550	6335	2053	2275	0.00902531	0.001192292
Ugt1a1	UDP glucuronosyltransferase 1 family, polypeptide A1	36	53	115	96	0.00823103	0.001199228
Cyr61	cysteine rich protein 61	421	554	203	275	0.00817434	0.001203213
Lrrn3	leucine rich repeat protein 3, neuronal	10	5	19	22	0.00799494	0.001207699
Ebp	3-beta-hydroxysteroid-Delta(8),Delta(7)-isomerase	1681	1862	3563	3969	0.00811885	0.001228517
Srm	spermidine synthase	2698	2828	1690	1955	0.0089982	0.001232508
Zrsr1	zinc finger (CCCH type), RNA-binding motif and serine/ar	12	26	37	39	0.00813369	0.001234684
Olfml3	olfactomedin 3	489	553	273	283	0.00897491	0.001244295
Ppargc1b	peroxisome proliferator-activated receptor gamma coactiv	89	109	35	54	0.00822489	0.001250643
Eif5a2	eukaryotic translation initiation factor 5A-2	1121	1454	556	656	0.00930434	0.001259527
Plin2	perilipin-2	4346	5511	7328	6841	0.01052075	0.001264061
Tnfrsf12a	tumor necrosis factor receptor superfamily member 12A, t	5261	5829	3747	4247	0.00839772	0.001275089
Znhit3	zinc finger HIT domain-containing protein 2	527	499	384	388	0.00844466	0.001292463
Mxd4	max dimerization protein 4	190	208	416	329	0.00854272	0.001294245
Gpr39	G protein-coupled receptor 39	87	93	162	153	0.00855852	0.001296521
Klhl24	kelch-like protein 24	257	331	640	550	0.01047383	0.001318916
Cdt1	Cdt1	1424	1548	627	935	0.00995113	0.001330762

Mdh1b	malate dehydrogenase 1B, NAD %28soluble%29	8	14	37	21	0.00871829	0.001343919
C1qtnf1	complement C1q tumor necrosis factor-related protein 1	13	20	33	34	0.00878858	0.001356627
Ppp1r15b	protein phosphatase 1, regulatory subunit 15b	1083	1280	1630	1568	0.00906154	0.001358218
Gcsh	glycine cleavage system H protein, mitochondrial	2046	2710	1376	1675	0.01009944	0.001372991
ZMYND12	zinc finger MYND domain-containing protein 12	66	85	268	204	0.01129847	0.001417398
Sfpq	splicing factor proline glutamine rich	5119	6052	3463	4309	0.01139042	0.001446818
Fn1	fibronectin 1, transcript variant 3	5209	5837	2480	2802	0.01309203	0.001461424
Fn1	fibronectin 1, transcript variant 2	5948	6625	2862	3206	0.01309285	0.001461546
Isg15	ubiquitin protein ISG15	11	6	18	27	0.00958472	0.00151152
Ly96	lymphocyte antigen 96	7	13	32	20	0.00959202	0.001512999
Timp1	metalloproteinase inhibitor 1	1212	1387	1961	1884	0.01129551	0.001517382
Tes	testin, transcript variant 1	765	826	1189	1320	0.01179943	0.001521392
C1ra	complement component 1, r subcomponent A	4548	4832	8216	6880	0.0121336	0.001535125
IFNGR2	interferon gamma receptor 2	371	428	649	714	0.01144134	0.001541428
Inha	inhibin alpha chain	76	85	185	123	0.01051495	0.00154786
Necap1	adaptin ear-binding coat-associated protein 1	1173	1389	2619	2414	0.01087053	0.00156088
TRAPPC4	trafficking protein particle complex subunit 4	995	1092	1556	1534	0.01245371	0.001578526
Gpt2	alanine aminotransferase 2	654	727	1908	1609	0.01017019	0.001604526
Pmf1	polyamine-modulated factor 1	418	467	168	239	0.01038296	0.001608924
Psap	prosaposin	10359	12346	16862	15817	0.01316497	0.001622112
P4ha3	proline 4-hydroxylase alpha polypeptide III	1112	1379	2007	1820	0.01083379	0.001626624
Rap2a	ras-related protein Rap-2a	134	175	99	119	0.01019078	0.001631225
Mroh7	HEAT repeat-containing protein 8	208	159	106	135	0.01019449	0.001631907
Mis18a	protein Mis18-alpha	435	430	217	273	0.0124269	0.001648873
NUDT22	nucleoside diphosphate-linked moiety X motif 22	275	330	551	524	0.01199079	0.001654985
PTRF	polymerase I and transcript release factor	5950	5885	3749	5036	0.01121105	0.001660984
KCTD17	BTB/POZ domain-containing protein KCTD17	569	608	943	963	0.01332212	0.001684833
Ahcy	S-adenosylhomocysteine hydrolase	5548	6305	3497	4197	0.01246462	0.001686966
Snx18	sorting nexin 18	1046	1235	1612	1455	0.01180014	0.001710996
Gmpr	GMP reductase 1	6	5	18	15	0.0106901	0.001731638
Ak3	GTP:AMP phosphotransferase, mitochondrial	3724	4547	2898	2835	0.01236413	0.001732727
Ran	GTP-binding nuclear protein Ran	16233	19080	11313	13641	0.0109118	0.001742897
S100a6	protein S100-A6	9996	12658	14487	14483	0.01133629	0.001746289
Klhl38	kelch protein 38	37	26	60	55	0.01077556	0.001748418
Nisch	nischarin	2204	2472	4688	4688	0.01542556	0.001762723
AK2	adenylate kinase 2, mitochondrial	1010	1171	784	839	0.01133259	0.00182263
Arl4d	ADP-ribosylation factor protein 4D	497	493	276	306	0.01156919	0.001822639
PDLIM2	PDZ and LIM domain protein 2, transcript variant 2	17	12	53	38	0.01148455	0.00182332
Acbd4	acyl-CoA-binding domain-containing protein 4	269	271	725	633	0.01482264	0.001848291
Il6st	interleukin 6 signal transducer	2314	2908	4606	3953	0.01457435	0.001855353
MAGEA10	melanoma-associated antigen 10	62	93	136	106	0.01132796	0.001862265
PIN1	peptidyl-prolyl cis-trans isomerase NIMA-interacting 1	2270	2409	1572	1839	0.01192611	0.001892979
Cbfb	core-binding factor subunit beta	573	669	373	462	0.01434918	0.001893983
Cacnb3	voltage-dependent L-type calcium channel subunit beta-3	447	383	796	900	0.01427398	0.001903902
Btg3	protein BTG3	314	366	555	574	0.01211682	0.001957607
Spsb1	SPRY domain-containing SOCS box protein 1	129	165	65	96	0.01194119	0.001961974
Mfsd3	major facilitator superfamily domain-containing protein 3	159	161	354	332	0.01353268	0.001962469
Slc10a3	solute carrier family 10, member 3	128	151	213	208	0.01372201	0.00200297
Unc80	unc-80 homolog %28C. elegans%29	6	5	19	14	0.01224368	0.002044923
Dpf3	zinc finger protein DPF3	22	27	10	12	0.0124038	0.002078217
Wnt4	protein Wnt-4	83	74	18	33	0.01311708	0.002078351
ICAM4	intercellular adhesion molecule 4	110	101	220	189	0.01399419	0.002123164
Slc9a9	solute carrier family 9, member 9	77	69	318	210	0.01723286	0.002136666
Canx	Canx	7168	8736	11314	12207	0.01406941	0.002148506
Fn1	fibronectin 1, transcript variant 1	6036	6710	2918	3256	0.01825553	0.002164768
Mars	methionyl-tRNA synthetase, cytoplasmic, transcript variar	1320	1436	3005	2662	0.01664264	0.00219758
Ccdc65	coiled-coil domain containing 65	10	10	26	21	0.01302639	0.00221014
ZNRF4	zinc/RING finger protein 4	8	16	24	29	0.01305691	0.002217669
Hsp90aa1	heat shock protein HSP 90-alpha	4064	5499	2943	3653	0.01394005	0.002253923
Acbd6	acyl-CoA-binding domain-containing protein 6	1220	1408	2020	1938	0.01686487	0.002266331
Mcm3	minichromosome maintenance deficient 3 (S. cerevisiae)	6709	7323	3459	4906	0.01517393	0.002277982
Dhx32	DEAH (Asp-Glu-Ala-His) box polypeptide 32	1377	1624	2703	2414	0.01667468	0.002291971
SMAGP	small cell adhesion glycoprotein	698	779	999	1018	0.01380411	0.002295224
Gpc1	glypican-1	7447	8089	4455	5974	0.01729541	0.002304698
Fgd4	FYVE, RhoGEF and PH domain containing 4	16	30	90	81	0.01348667	0.00230481
Chchd4	mitochondrial intermembrane space import and assembly	982	1169	749	816	0.0143086	0.002304843
Thra	thyroid hormone receptor alpha, transcript variant 3	520	637	980	974	0.01786444	0.002307427
Klf10	Krueppel factor 10	254	308	132	148	0.01369653	0.002310994
Dclk1	doublecortin kinase 1, transcript variant 3	44	64	20	21	0.01372885	0.002320156
Dclk1	doublecortin kinase 1, transcript variant 5	44	64	20	21	0.01373008	0.002320587

Dclk1	doublecortin kinase 1, transcript variant 1	44	64	20	21	0.0137317	0.002320989
Hsd17b13	17-beta-hydroxysteroid dehydrogenase 13, transcript vari	13	6	38	19	0.01354675	0.002323941
Thra	thyroid hormone receptor alpha, transcript variant 2	585	693	1057	1054	0.01815813	0.002335865
Csrp3	cysteine and glycine-rich protein 3	6	4	11	21	0.01360753	0.00233599
Dhodh	dihydroorotate dehydrogenase	1260	1369	619	720	0.01496917	0.002359572
Hsbp1	heat shock factor-binding protein 1	849	1023	1338	1371	0.01400797	0.002371987
Hspd1	heat shock protein 1 (chaperonin)	25600	31901	19212	22909	0.01750702	0.002382029
Leprot	leptin receptor gene-related protein	852	934	1320	1384	0.01430211	0.002389843
NPM1	nucleophosmin	12887	17507	9784	10952	0.01747348	0.002392243
Asl	argininosuccinate lyase, transcript variant 1	6	10	21	19	0.01393968	0.002410743
Asl	argininosuccinate lyase, transcript variant 2	6	10	21	19	0.01393968	0.00241082
Asl	argininosuccinate lyase, transcript variant 3	6	10	21	19	0.0139408	0.002411133
Nudt18	nucleoside diphosphate-linked moiety X motif 18	143	183	319	304	0.01421932	0.002423357
C1GALT1C1	C1GALT1-specific chaperone 1	529	762	851	831	0.0141055	0.002447668
Yme1l1	YME1 1 %28S. cerevisiae%29	2656	3424	5281	5032	0.01690346	0.002503219
Cpeb3	cytoplasmic polyadenylation element-binding protein 3	72	71	191	203	0.01475277	0.002504474
Gstm1	glutathione S-transferase Y1	3560	3711	6052	5997	0.01440388	0.002512159
RMI1	recQ-mediated genome instability protein 2	379	443	183	264	0.01442332	0.002518899
Pa2g4	proliferation-associated protein 2G4	11112	13506	7618	8526	0.01665787	0.00255865
Lyrm2	LYR motif-containing protein 2	219	273	382	328	0.01680181	0.002590455
Pcdhgb1	protocadherin gamma-B1	432	498	590	591	0.01475642	0.002593044
Zfp703	zinc finger protein 703	594	614	744	797	0.01483864	0.002611536
Npc1	Npc1	1298	1633	430	504	0.01781096	0.002618717
FBXO5	F-box only protein 5	680	840	400	496	0.01661937	0.002620872
NSDHL	sterol-4-alpha-carboxylate 3-dehydrogenase, decarboxyla	1586	1732	3902	4162	0.01501997	0.002652145
Vamp2	vesicle-associated membrane protein 2	73	96	164	153	0.01626355	0.002661919
St14	suppression of tumorigenicity 14 %28colon carcinoma%2	140	151	587	582	0.0175425	0.002665497
Thra	thyroid hormone receptor alpha	133	171	277	269	0.01620227	0.002672554
Abhd15	abhydrolase domain containing 15	9	12	21	26	0.01524948	0.002704103
MKRN1	E3 ubiquitin-protein ligase makorin-1	642	832	1274	1387	0.01543741	0.002722521
Tubb2c	Tubb2c	13663	16492	8406	12942	0.01761611	0.002735484
Slc35a4	probable UDP-sugar transporter protein SLC35A4	406	430	308	346	0.01540735	0.002741046
Flot2	flotillin-2, transcript variant 1	1491	1726	3247	3187	0.01564604	0.002751712
Kdelr3	ER lumen protein retaining receptor 3	972	1236	1557	1534	0.01669157	0.002755904
Nuak1	NUAK family, SNF1 kinase, 1	79	95	53	64	0.01551782	0.002766093
Dcakd	dephospho-CoA kinase domain-containing protein	3327	3428	2415	2613	0.01775606	0.002796697
Bean1	protein BEAN1	50	46	11	20	0.01570224	0.002803092
MED7	mediator of RNA polymerase II transcription subunit 7	140	154	222	188	0.01569163	0.002806564
Ror1	receptor tyrosine kinase orphan receptor 1	298	323	609	634	0.01891184	0.002811494
FBXO36	F-box only protein 36	8	4	21	14	0.01574245	0.00281981
Lypla1	lysophospholipase protein 1	222	243	557	432	0.01686573	0.002855485
PDIA6	protein disulfide-isomerase A6	12228	13749	17213	19723	0.01966186	0.002859223
H2-K1	h-2 class I histocompatibility antigen, K-B alpha chain	2037	2253	2887	2798	0.01819456	0.002874971
UBIAD1	ubiA prenyltransferase domain-containing protein 1	956	1076	718	778	0.0160818	0.002893231
FBXW2	F-box/WD repeat-containing protein 2	1277	1565	2224	2221	0.0176598	0.002898057
Cpne9	copine-9	17	24	7	10	0.01612891	0.002906265
Ttf2	transcription termination factor, RNA polymerase II	1535	1666	470	580	0.02123392	0.002921505
Cct5	T-complex protein 1 subunit epsilon, transcript variant 1	13144	15916	9826	10853	0.01826783	0.002946097
Cct5	T-complex protein 1 subunit epsilon, transcript variant 2	13087	15868	9793	10827	0.01826811	0.002946198
Zfp664	zinc finger protein 664	573	657	827	751	0.0163952	0.002971362
Lpin1	Lpin1	60	67	299	260	0.02053121	0.003004127
Sox8	SRY-box containing gene 8	25	29	8	17	0.01656305	0.003011373
NUDT6	nucleoside diphosphate-linked moiety X motif 6	13	20	51	44	0.01886758	0.003098061
STAR	steroidogenic acute regulatory protein, mitochondrial	29	26	11	15	0.01699219	0.003112264
FAM43A	protein FAM43A	146	164	214	205	0.01713602	0.003146883
Il25	interleukin-25	37	26	74	74	0.01793304	0.003149048
Abcc5	ATP-binding cassette, sub-family C %28CFTR%2FMRP%	205	307	406	419	0.0217757	0.003151615
CPN1	carboxypeptidase N catalytic chain	19	15	8	4	0.01722748	0.003169444
Cdkn1a	cyclin-dependent kinase inhibitor 1A	922	1143	1771	1949	0.01736181	0.003198245
Flot2	flotillin-2, transcript variant 2	1442	1689	3169	3083	0.01775088	0.003227982
RHNO1	RAD9, RAD1, HUS1-interacting nuclear orphan protein	927	1124	731	843	0.01928115	0.0032378
Ccdc125	coiled-coil domain containing 125	29	37	52	56	0.01763231	0.003263597
Igsf9	immunoglobulin superfamily, member 9	5	6	15	16	0.01769291	0.003280087
RASSF8	Ras association (RalGDS/AF-6) domain family (N-termina	1368	1567	749	911	0.01844105	0.003321208
SDR39U1	epimerase family protein SDR39U1	30	29	45	56	0.01785598	0.003322626
Slc52a3	riboflavin transporter 2, transcript variant 1	1008	1126	1578	1731	0.01824432	0.003367642
Hmgb2	high mobility group protein B2	2001	2573	1608	1920	0.02243802	0.003386717
Slc52a3	riboflavin transporter 2, transcript variant 2	316	359	537	564	0.01946463	0.003502996
Gtdc1	glycosyltransferase domain containing 1	79	95	294	273	0.02331567	0.003570124
THOC2	THO complex subunit 2	312	403	477	446	0.0189749	0.003590285

P2RY1	P2Y purinoceptor 1	25	28	12	14	0.01909482	0.003619503
Man2b1	mannosidase 2, alpha B1	815	912	2344	1905	0.02510188	0.003670868
Gstt2	glutathione S-transferase theta-2	218	240	501	409	0.02402471	0.003692539
Pawr	PRKC apoptosis WT1 regulator protein	649	808	1460	1366	0.02054557	0.003695032
Slc25a45	solute carrier family 25 member 45	56	59	151	115	0.02098641	0.003752367
Gba	glucosidase, beta, acid	745	866	1689	1406	0.02246967	0.003791812
RDH14	retinol dehydrogenase 14	232	237	415	393	0.01989474	0.0038137
Gdpd5	glycerophosphodiester phosphodiesterase domain-contai	66	65	15	33	0.02367562	0.003841916
B3GALT4	beta-1,3-galactosyltransferase 4	120	127	90	87	0.02009618	0.003860674
CCNG1	cyclin-G1	1018	1529	2250	2222	0.02181768	0.003864484
Elovl4	elongation of very long chain fatty acids protein 4	80	86	27	20	0.02245484	0.003876768
Ets2	protein C-ets-2	560	596	1201	1153	0.02038062	0.003881701
Egr1	early growth response protein 1	703	838	1027	928	0.02022275	0.003891189
Ruvbl1	ruvB-like 1	5176	5853	3625	4282	0.02813478	0.003977484
Slc44a1	solute carrier family 44, member 1	1687	2031	3964	3989	0.02242711	0.004069666
Sdhaf2	succinate dehydrogenase assembly factor 2, mitochondri	1795	2075	3048	2648	0.02470619	0.004130444
DUSP3	dual specificity protein phosphatase 3	112	148	208	194	0.02315778	0.004197392
Lfng	beta-1,3-N-acetylglucosaminyltransferase lunatic fringe	81	86	17	23	0.02663447	0.004267248
Srrt	Srrt	1806	2041	1087	1435	0.02564637	0.004321652
Jup	junction plakoglobin	24	27	175	150	0.02257496	0.004356574
Iscu	iron-sulfur cluster assembly enzyme ISCU, mitochondrial	790	857	1162	1191	0.02810241	0.004414211
Acot6	acyl-CoA thioesterase 6	209	172	260	272	0.02239378	0.004414214
Ccdc103	coiled-coil domain-containing protein 103	47	45	97	98	0.02732748	0.004418284
Srsf1	serine%2Farginine-rich splicing factor 1	7063	8513	6054	6319	0.02271358	0.004457982
Rgs17	regulator of G-protein signaling 17	156	210	582	627	0.02430943	0.004501825
C3AR1	C3a anaphylatoxin chemotactic receptor	168	208	144	138	0.02286143	0.004531708
Scn9a	sodium channel, voltage-gated, type IX, alpha, transcript	9	21	34	28	0.02286851	0.004533925
Scn9a	sodium channel, voltage-gated, type IX, alpha, transcript	9	21	34	28	0.02286851	0.00453428
Scn9a	sodium channel, voltage-gated, type IX, alpha, transcript	9	21	34	28	0.02286911	0.004534594
Pfn1	profilin-1	5765	6153	4452	5178	0.02570081	0.004567645
Chml	rab proteins geranylgeranyltransferase component A 2	614	779	889	853	0.02311595	0.004597321
Sfrp4	secreted frizzled-related protein 4	215	274	510	469	0.02757309	0.004611507
NME1	nucleoside diphosphate kinase A, transcript variant 1	4906	5710	3747	4043	0.02673851	0.004618504
Myo1g	myosin IG	19	15	7	6	0.02372201	0.004757735
Pex11g	peroxisomal membrane protein 11C	38	50	116	104	0.0273361	0.004770121
Mrps6	28S ribosomal protein S6, mitochondrial	284	317	206	221	0.0261912	0.004770574
BATF2	basic leucine zipper transcriptional factor ATF-like 2	48	62	87	78	0.02381448	0.004782895
Ipo5	importin-5	10587	13145	6696	7980	0.03125941	0.004826275
RAD18	E3 ubiquitin-protein ligase RAD18	247	322	185	246	0.024116	0.004861026
Utp14b	UTP14, U3 small nucleolar ribonucleoprotein, homolog B	369	417	508	542	0.02764347	0.004869939
Klhl36	kelch 36 %28Drosophila%29	139	151	231	251	0.02495169	0.004893445
Gtpbp2	GTP binding protein 2	256	300	728	589	0.0304197	0.004894468
Mpp1	membrane protein, palmitoylated	1067	1311	2160	2093	0.02670118	0.004916644
Pcbp2	poly(rC)-binding protein 2	5959	6626	4958	5458	0.02762111	0.004925782
MUM1L1	PWWP domain-containing protein MUM1L1	1109	1397	990	1104	0.02531172	0.004927341
Rps15	40S ribosomal protein S15	15312	16742	10551	11669	0.02555552	0.004934378
Zfand3	AN1-type zinc finger protein 3	424	533	763	717	0.03253863	0.005071467
